# Single-cell RNA-seq analysis unveils a prevalent epithelial/mesenchymal hybrid state during mouse organogenesis

**DOI:** 10.1186/s13059-018-1416-2

**Published:** 2018-03-14

**Authors:** Ji Dong, Yuqiong Hu, Xiaoying Fan, Xinglong Wu, Yunuo Mao, Boqiang Hu, Hongshan Guo, Lu Wen, Fuchou Tang

**Affiliations:** 1 Beijing Advanced Innovation Center for Genomics (ICG), Ministry of Education Key Laboratory of Cell Proliferation and Differentiation, College of Life Sciences, Peking University, Beijing, 100871 People’s Republic of China; 2 Biomedical Institute for Pioneering Investigation via Convergence, College of Life Sciences, Peking University, Beijing, 100871 People’s Republic of China; 3 Peking-Tsinghua Center for Life Sciences, Peking University, Beijing, 100871 People’s Republic of China

**Keywords:** Single-cell RNA-seq, Organogenesis, Interactions between mesenchyme and epithelium, Epithelial/mesenchymal hybrid state

## Abstract

**Background:**

Organogenesis is crucial for proper organ formation during mammalian embryonic development. However, the similarities and shared features between different organs and the cellular heterogeneity during this process at single-cell resolution remain elusive.

**Results:**

We perform single-cell RNA sequencing analysis of 1916 individual cells from eight organs and tissues of E9.5 to E11.5 mouse embryos, namely, the forebrain, hindbrain, skin, heart, somite, lung, liver, and intestine. Based on the regulatory activities rather than the expression patterns, all cells analyzed can be well classified into four major groups with epithelial, mesodermal, hematopoietic, and neuronal identities. For different organs within the same group, the similarities and differences of their features and developmental paths are revealed and reconstructed.

**Conclusions:**

We identify mutual interactions between epithelial and mesenchymal cells and detect epithelial cells with prevalent mesenchymal features during organogenesis, which are similar to the features of intermediate epithelial/mesenchymal cells during tumorigenesis. The comprehensive transcriptome at single-cell resolution profiled in our study paves the way for future mechanistic studies of the gene-regulatory networks governing mammalian organogenesis.

**Electronic supplementary material:**

The online version of this article (10.1186/s13059-018-1416-2) contains supplementary material, which is available to authorized users.

## Background

During mammalian embryonic development, organogenesis is a crucial process leading to the diversification of different organs and cell types. Organogenesis starts when the neural tube is formed and the mesodermal cells are segmented into somites. The onset of mouse organogenesis begins at approximately E8.0, and the buds of all major organs are essentially formed at E9.5. Along with development, interactions between epithelium and mesenchyme are crucial for the proper development of all organs with epithelial parenchyma [1]. Through interactions with the mesenchyme, epithelial identity is induced and specified [2, 3]. Another important cellular mechanism characterizing embryonic development is the epithelial-mesenchymal transition (EMT), which is involved in many developmental processes (for instance, gastrulation, neural crest development, and somite dissociation) [4–6]. Through EMT, cells transit from the epithelial state to the mesenchymal state, acquiring a migratory and invasive feature. However, instead of a single binary switch between the full epithelial and full mesenchymal states, EMT is a process that consists of multiple and dynamic intermediate phases. Cells are able to linger in intermediate stages and frequently present an epithelial/mesenchymal (E/M) hybrid state [7].

Recently, the transcript diversity from gastrulation through organogenesis of mammalian embryos has been studied by bulk-cell RNA-seq [8–11]. However, few studies have focused on cellular heterogeneity among organs at single-cell resolution, especially during the early stages of organogenesis. Although there are some single-cell RNA-seq data of organs from different groups’ efforts to dissect the transcriptome of mouse organogenesis [12–15], the organs used were not from the same stages or the same embryos, resulting in sampling and technical variations among studies. Therefore, a parallel analysis of single cells from different organs within the same mouse embryo is more appropriate for eliminating the batch effect, sampling variation, and other technological biases.

Many studies based on single-cell RNA-seq used gene expression matrices to perform clustering analyses [16–18]. Generally, genes that contribute to cellular phenotypes can be divided into two classes: “realizer” genes, such as those encoding enzymes, cytoskeletal proteins, etc., and regulatory genes, including those encoding transcription factors (TFs) and co-factors [19–21]. Realizer genes directly maintain the cellular physiological phenotype, so their expression pattern more likely reflects functional similarities, while regulatory genes regulate realizer genes to affect the cellular phenotype, which makes them more specific and crucial to the identity and function of the cells [22].

In this study, we mainly used regulatory activity-based methods, assisted by expression patterns, to reveal the evolutionary or developmental relationships of various organs and cell types during mouse organogenesis. Specifically, we analyzed 1916 individual cells from eight organs or tissues of seven E9.5–E11.5 mouse embryos. Our results reveal the expression patterns and developmental paths of distinct organs and many interactions between distinct cell types. Additionally, we detect an E/M hybrid state in epithelial cells during organogenesis, whose molecular features are shared by tumor cells during tumorigenesis. Thus, our work provides a single-cell resolution resource for the transcriptomic features of early mouse organogenesis and paves the way for future functional studies of lineage formation and differentiation for each major organ in mammals.

## Results

### Developmental landscape of mouse organogenesis

**Fig. 1:**
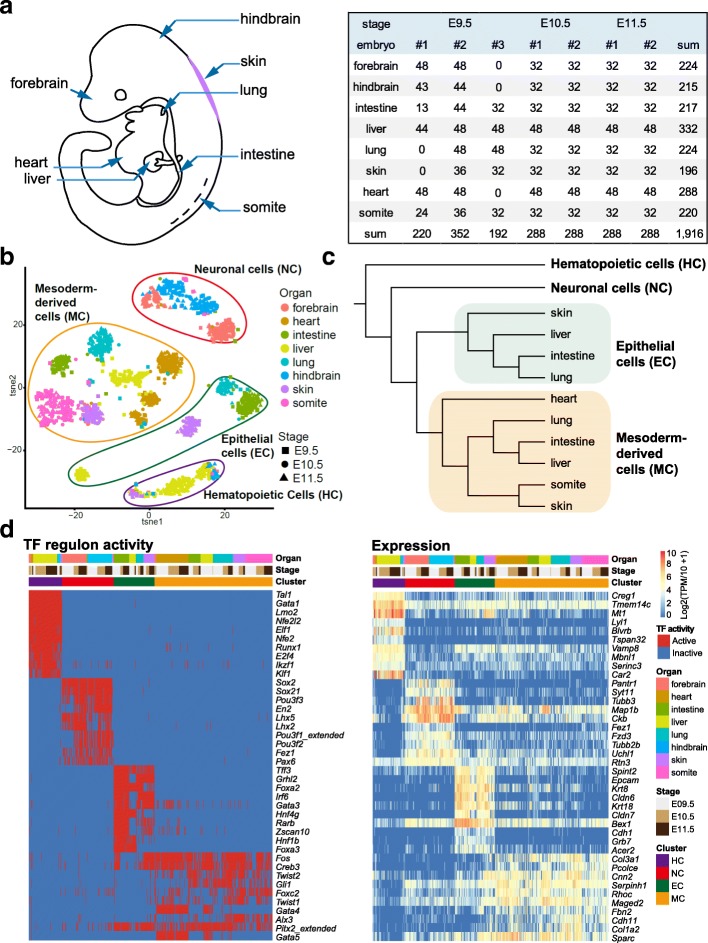
Global patterns of single-cell expression profiles and the identification of cell types. **a** Schematic of the sampling position (*left*) and sampling information (*right*) of each mouse organ and tissue. **b** Regulon matrix-based t-distributed stochastic neighbor embedding (t-SNE) plot showing the origin and embryonic stage of the cells. Organ types are indicated by colors, and developmental stages are indicated by shapes. The major groups identified are circled and annotated. **c** Hierarchical clustering through the regulon matrix showing the relationships of cells sampled from different organs and major groups identified by the regulon matrix. **d** Heatmaps showing the top 10 group-specific transcription factors (*TFs*, *left*) and differentially expressed genes (*DEGs*, *right*) of each major group. The color key from *blue* to *red* indicates low to high gene expression or TF activity, respectively

To study the organogenesis of multiple organs at single-cell resolution, we sequenced 1916 single cells from eight major organs and tissues, namely, the forebrain, hindbrain, skin, heart, somite, intestine, liver, and lung, from three E9.5, two E10.5, and two E11.5 mouse embryos (Fig. 1a). After stringent filtering, 1819 single cells with high quality were obtained to conduct subsequent analyses. On average, 6361 genes and 0.43 million unique molecular identifier (UMI) transcripts were detected in each individual cell (Additional file 1: Figure S1a). Cells sampled from different embryos were mixed well, and no batch effect was detected, as shown in the t-distributed stochastic neighbor embedding (t-SNE) plot (Fig. 1b; Additional file 1: Figure S1c and S1d). We also conducted a saturation analysis to make sure that the sequencing depth was sufficient for the subsequent analyses. As shown in Additional file 1: Figure S1b, from just half of the original reads, we could still detect 90% of the expressed genes compared with using all of the original reads. This indicates that the current sequencing depth was sufficient for the subsequent analyses.

To explore the evolutionary or developmental relationships among organs, we used SCENIC [23] to map gene-regulatory networks (GRNs) from our single-cell RNA-seq data. SCENIC is an algorithm that can reconstruct GRNs and identify stable cell states (see Methods). We performed an unsupervised clustering analysis adjusted by the random forest algorithm using a binary regulon activity matrix generated by SCENIC (we will call this the regulon matrix for convenience) and a gene expression matrix.

Four major groups were determined through the regulon matrix, and their differentially expressed genes (DEGs) were also identified (Fig. 1c and d). Based on the top TFs, gene markers, and enriched terms (Additional file 1: Figure S1e), we assigned these four major groups as hematopoietic cells, where TFs such as *Gata1*, *Tal1*, *Runx1*, and *Klf1* were specifically active; neuronal cells, which specifically activate TFs such as *Sox2*, *Sox21*, and *Pax6*; epithelial cells, exhibiting high expression of genes that are crucial for epithelial cells, such as *Epcam*, *Cldn6*, *Cldn7*, and *Cdh1*; and mesoderm-derived cells, expressing marker genes including *Col3a1*, *Pcolce*, and *Cdh11*. As expected, most of the hematopoietic cells were sampled from the liver, because during this developmental period, erythro-myeloid progenitors (EMPs) seed the fetal liver and execute hematopoiesis. Neuronal cells were mainly composed of cells sampled from forebrain and hindbrain, except several cells from the somites and intestine. Epithelial cells were exclusively composed of cells sampled from three endoderm organs (intestine, liver, and lung) and one ectoderm organ (skin). Mesenchymal cells of these four organs were included in the mesoderm-derived cell group, as were cells of mesoderm organs (heart and somites).

On the other hand, expression matrix-based clustering generated a similar pattern as revealed in the t-SNE and the hierarchy tree, indicating the accuracy of clustering by the regulon matrix (Additional file 1: Figure S1c). However, the hierarchy clustering based on the regulon matrix was more reasonable in some details compared with that based on the expression matrix. For example, in the hierarchy tree constructed by the expression matrix, some heart cells (cardiomyocytes) were rooted outside the epithelial cells and mesoderm-derived cells, while all mesoderm-derived cells were in the mesoderm-derived cell group when using the regulon matrix. Thus, as mentioned in the Background, instead of evolutionary or developmental relatedness, hierarchy clustering based on the expression matrix more likely reflected functional similarities. Given that, we used the developmental hierarchy constructed from the regulon matrix to carry out the subsequent analyses, and the expression matrix was important for complementarity.

### Development of epithelial cells and interactions between mesenchyme and epithelium

**Fig. 2:**
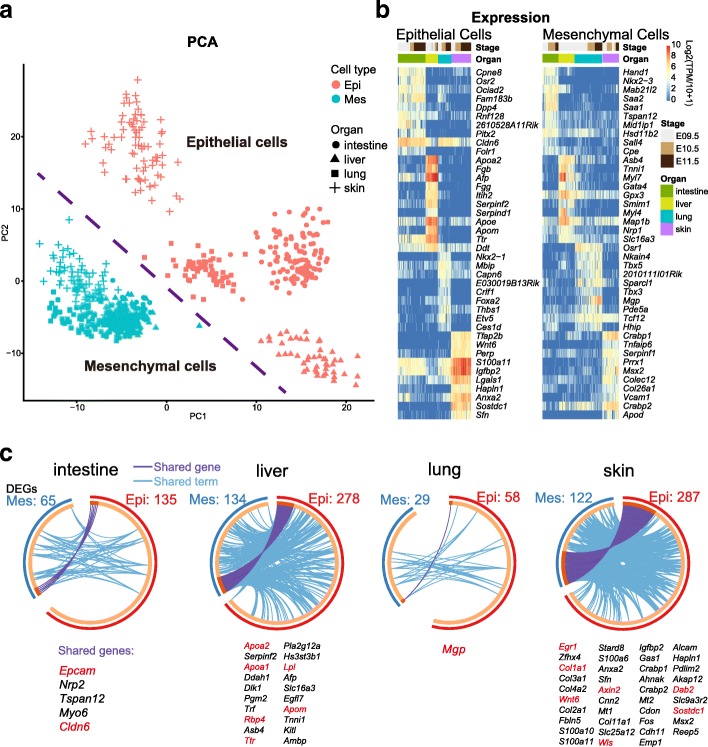
Interaction between epithelial and mesenchymal cells sampled from intestine, liver, lung, and skin. **a** Principal component analysis (*PCA*) of epithelial and mesenchymal cells. Cell types are indicated by colors, and organ types are indicated by shapes. **b** Heatmaps showing the top 10 DEGs of epithelial (*left*) and mesenchymal (*right*) cells sampled from each organ. The color key from *blue* to *red* indicates low to high gene expression, respectively. **c** Circos plots showing interaction between epithelial and mesenchymal cells. The shared genes are linked by *purple lines*, and the different genes falling into the same term are linked by *blue lines*

In our data, we captured epithelial cells from four organs composed of epithelial parenchyma, namely, intestine, liver, lung, and skin, as well as their mesenchymal counterparts. The first two axes of our principal component analysis (PCA) well separated epithelial and mesenchymal cells as distinct cell types (Fig. 2a). These data provided a valuable chance to identify the interactions between epithelium and mesenchyme, one of the fundamental developmental mechanisms responsible for the development of the majority of organs [1].

We reasoned that the organ-specific DEGs of either epithelial or mesenchymal cells would represent specific requirements for the development of a certain organ. Moreover, for a certain organ, if some of the epithelial DEGs and mesenchymal DEGs participated in the same biological processes, these genes and biological processes would provide valuable information on the putative interaction between epithelial and mesenchymal cells. Thus, we first performed a differential expression analysis to obtain the organ-specific DEGs for epithelial and mesenchymal cells (Fig. 2b; Additional file 1: Figure S2a). The top organ-specific TFs also supported their identities (Additional file 1: Figure S2b). Then, we used the Meta-analysis workflow in Metascape [24] to combine these organ-specific DEGs of epithelial and mesenchymal cells to identify the shared pathways in which they participated. Both cell type-specific and shared terms were enriched for each organ, except skin, whose terms were nearly all mutual for epithelial and mesenchymal cells (Additional file 1: Figure S2c). Specifically, in the intestine, the shared terms included digestive tract development and regulation of cellular component movement; in the liver, retinoid metabolism and transport and lipoprotein metabolism; in the lungs, tube development and lung development; and in the skin, Wnt signaling pathway, cell substrate adhesion, and others. Actually, the shared DEGs between epithelial and mesenchymal cells had already provided clear clues regarding their mutual regulation (Fig. 2c). For example, the shared DEGs in the intestine, *Epcam* and *Cldn6*, are both related to cell adhesion; in the liver, *Apoa1/2*, *Lpl*, *Rbp4*, *Ttr*, and *Apom* are related to retinoid metabolism and transport; in the lung, *Mgp* is involved in bone mineralization, which is important for tube development; and in the skin, *Axin2*, *Col1a1*, *Dab2*, *Egr1*, *Wnt6*, *Sostdc1*, and *Wls* are related to the Wnt signaling pathway.

**Fig. 3:**
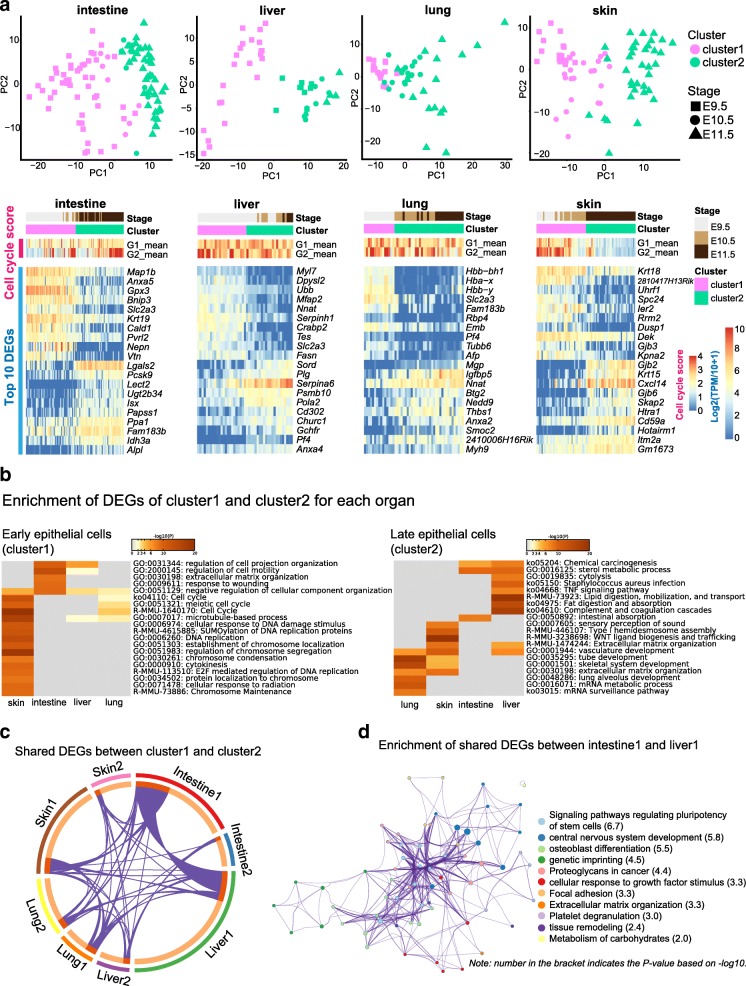
Development of epithelial cells sampled from intestine, liver, lung, and skin. **a** Principal component analysis (PCA) of epithelial cells sampled from different organs (*top*). Clusters are indicated by colors, and developmental stages are indicated by shapes. Heatmaps showing the top 10 DEGs of each cluster in different organs (*bottom*). The color key from *blue* to *red* indicates low to high gene expression, respectively. **b** Heatmaps showing enrichment of DEGs of all early epithelial cells (*cluster1*, *left*) and all late epithelial cells (*cluster2*, *right*). The color key from *gray* to *brown* indicates high to low *P* values, respectively. **c** Circos plots showing shared DEGs among clusters of epithelial cells. The shared genes are linked by *purple lines*. **d** Enrichment network of shared DEGs between intestine1 and liver1. Each term is indicated by a *circular node*. The number of input genes falling into that term is represented by the circle size and the cluster identities are represented by colors. *P* values based on –log_10_ are given in the brackets

The preceding analyses were based on the whole organ, which ignored the developmental factors. Thus, we next investigated the molecular-developmental features of these organs. Because of the limited resolution of the regulon matrix, we used the expression matrix to conduct further unsupervised clustering for epithelial cells of each organ. Epithelial cells in each organ were split into two subclusters, showing their developmental order (Fig. 3a). We also performedPCA, and the first axis of the PCA ordered the cells according to their developmental time in each of the four organs (Fig. 3a). Meanwhile, the PCA also ordered the subclusters and confirmed the accuracy of the further clustering. We thus named them cluster 1 (early epithelial cells) and cluster 2 (late epithelial cells). Apparently, during these developmental stages, epithelial cells continuously developed.

We wondered whether these organs possessed similar developmental patterns. To explain the organ-specific developmental direction, we used the Meta-analysis workflow to combine DEGs between cluster 1 and cluster 2 (Fig. 3b). Intestine and liver early epithelial cells showed characteristics of movement, while lung and skin early epithelial cells shared several terms related to the cell cycle. Interestingly, epithelial cells of intestine cluster 1 and liver cluster 1 shared many genes, indicating similar developmental patterns at early stages (Fig. 3c). In addition, these genes were enriched in terms related to pluripotency and cell adhesion (Fig. 3d). On the other hand, late epithelial cells (cluster 2) of these four organs exhibited organ-specific developmental patterns (Fig. 3b). For example, DEGs of intestine were enriched for terms related to intestine absorption, fat and lipid digestion in liver, lung alveolus development in lung, and sensory perception of sound in skin.

### Prevalent epithelial/mesenchymal hybrid state in epithelial cells during mouse organogenesis

**Fig. 4:**
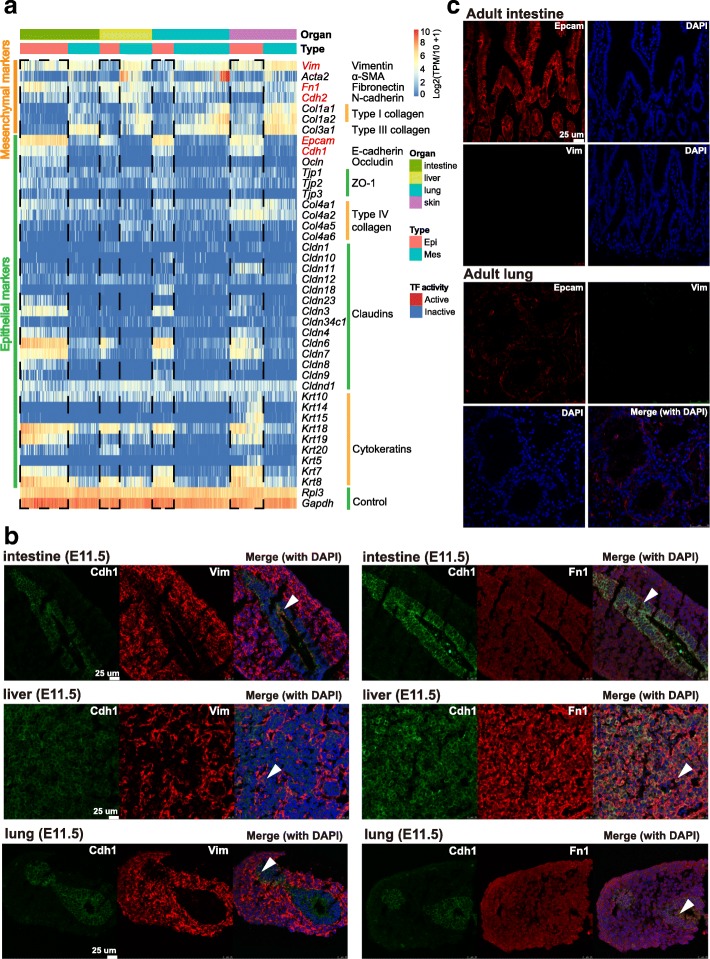
Prevalent epithelial/mesenchymal hybrid state in epithelial cells during organogenesis. **a** Heatmap showing the representative epithelial and mesenchymal markers in epithelial and mesenchymal cells. The color key from *blue* to *red* indicates low to high gene expression, respectively. **b** Immunostaining of Cdh1, Vim, and Fn1 in E11.5 intestine, liver, and lung. The *white arrow* indicates potential co-expression of Cdh1 and Vim. **c** Immunostaining for Epcam and Vim in adult intestine and lung

It is well known that the transition between epithelial and mesenchymal features plays an important role in embryonic development [6, 7, 25]. We asked whether this critical cellular mechanism was also involved in the development of the epithelial cells. Indeed, we found a prevalent E/M hybrid state in the epithelial cells of all four epithelial parenchyma organs. As revealed in Fig. 4a, epithelial cells with high expression of epithelial markers, such as *Epcam*, *Cdh1*, claudins, and cytokeratins, also highly expressed *Vim*, *Fn1*, and *Sparc* (*Sparc* in right panel of Fig. 1d), genes with mesenchymal characteristics. This phenomenon was also validated by immunostaining: both the mesenchymal markers Vim and Fn1 were co-expressed with Cdh1, a typical epithelial marker (Fig. 4b; Additional file 1: Figure S3a). The immunostaining of Fn1 was strong in intestine, liver, and lung epithelial cells (Cdh1-positive cells in Fig. 4b), while in adult liver the immunostaining of Fn1 was restricted to specific cells (Additional file 1: Figure S3b), confirming that the immunostaining of Fn1 in Fig. 4b was accurate.

**Fig. 5:**
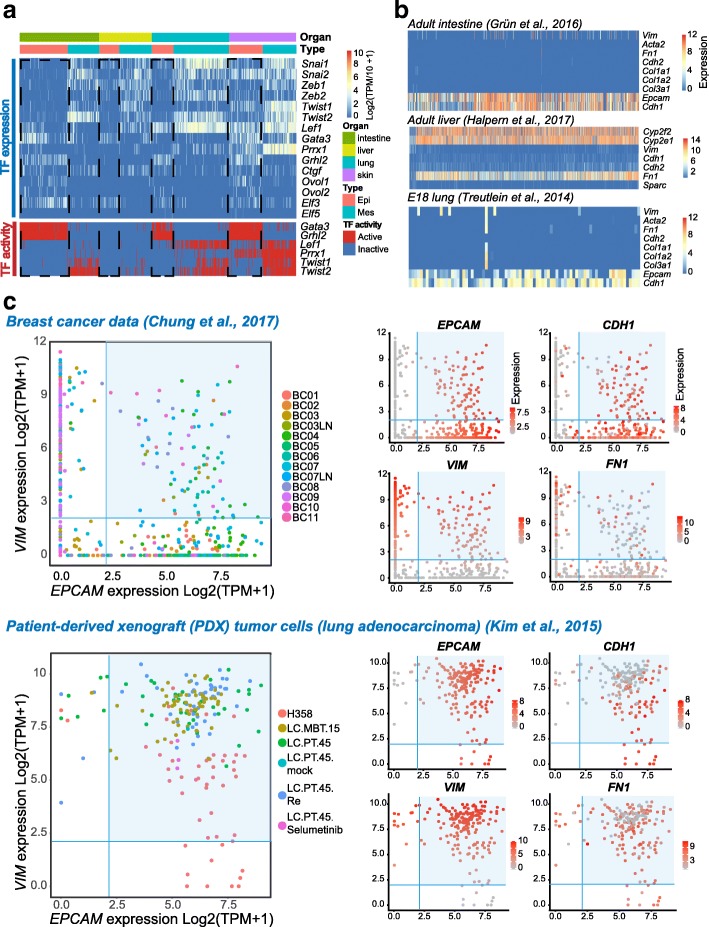
Expression pattern of representative EMT-related TFs and expression pattern of representative markers in late-stage or adult organs and two carcinoma datasets. **a** Heatmaps showing the representative EMT-related TFs in epithelial and mesenchymal cells. The color key from *blue* to *red* indicates low to high gene expression or TF activity, respectively. **b** Heatmaps showing the representative markers in adult intestine, adult liver, and E18 lung [15, 30, 31]. **c** Scatterplots showing the expression of representative markers in two carcinoma datasets [32, 33]. Cells sampled from different sources are represented by colors at the first plot of each dataset. For all plots, the *x*-axis measures the expression value of *EPCAM*, and the *y*-axis measures the expression value of *VIM*. Cells whose expression values of both *EPCAM* and *VIM* exceed 2 are shadowed in *blue*, indicating the potential E/M hybrid state. The expression values of representative markers are indicated by colors. The color key from *gray* to *red* indicates low to high gene expression, respectively

Surprisingly, the classical EMT-inducting TFs (*Snai1/2*, *Zeb1/2*, and *Twist1/2*) were barely expressed in these epithelial cells, except for skin, where *Snai2* and *Twist1* were expressed at moderate levels in some cells (Fig. 5a). In addition, when we checked the TF activity, *Twist1/2* were not active in these cells (Fig. 5a). This was understandable, since these TFs were well known for their ability to repress epithelial features [7]. However, these epithelial cells still expressed epithelial markers. Thus, the regulatory mechanism governing this hybrid state might be different from classical EMT mechanisms. We noticed that a TF, *Grhl2*, which protects the epithelial phenotype [7, 26], was active in the epithelial cells of intestine, lung, and skin. GATA-binding protein 3 (*GATA3*) was also active, and previous evidence showed that it could inhibit the miR-200 family and that it promotes EMT [5]. *Ovol* genes were not highly expressed across epithelia, though they were shown to stabilize the hybrid E/M phenotype [27, 28]. *Elf3* was expressed in several epithelial cells, while *Elf5* was not. Both *Elf3* and *Elf5* were shown to be negative regulators of EMT [29]. In contrast, in the datasets of adult or mature epithelial cells from intestine, liver, and E18 lung, they barely presented an E/M hybrid state (Fig. 5b) [15, 30, 31]. As a control, the immunostaining showed that the Epcam-positive epithelial cells of adult intestine and lung were negative for Vim as expected (Fig. 4c). This meant that the observed E/M hybrid state only occurred at particular developmental stages in organs with epithelial parenchyma.

Since the transition between epithelial and mesenchymal features has been associated with the cancer metastatic cascade, we asked whether this kind of E/M hybrid state was also prevalent in cancer cells. In two types of carcinoma datasets, breast and lung cancers [32, 33], the E/M hybrid state also existed (Fig. 5c). Given the similar E/M hybrid pattern in developing epithelial organs and carcinomas, we assumed that organs with carcinomas might not create a novel mechanism to perform metastasis; instead, they just utilize the mechanism that already exists during normal embryonic development. Therefore, it was crucial to understand what happens during embryonic organogenesis and find the similarities and differences in the E/M hybrid states between carcinoma metastasis and embryonic organogenesis.

**Fig. 6:**
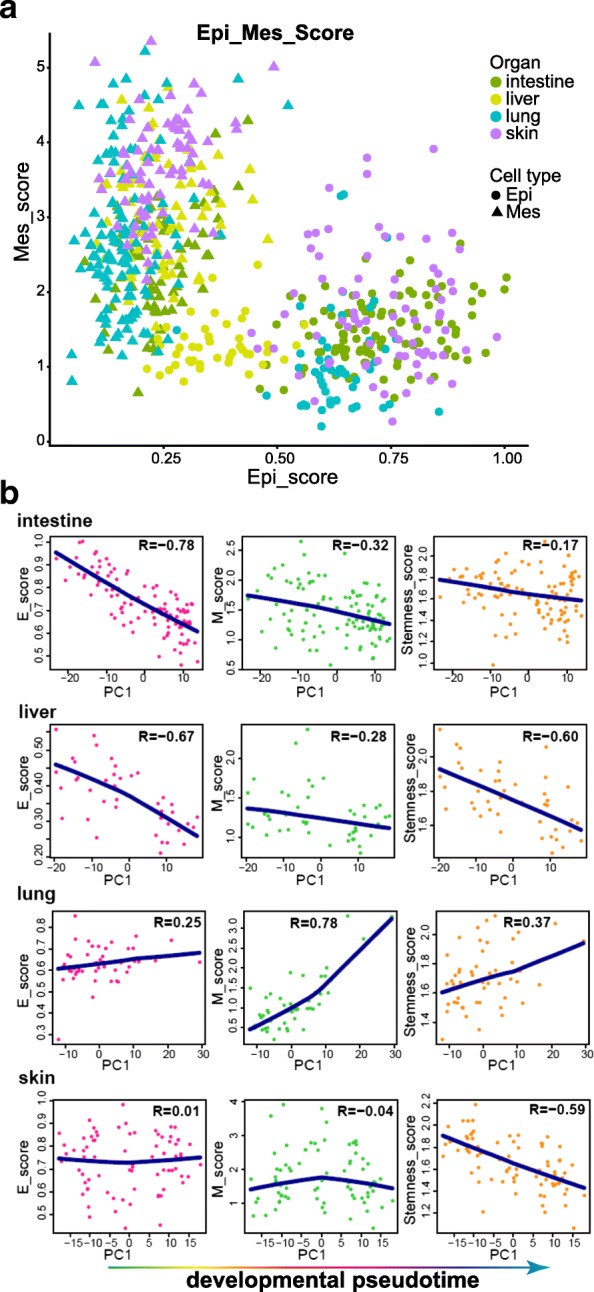
Epithelial, mesenchymal, and stemness scores of cells sampled from intestine, liver, lung, and skin. **a** Scatterplots showing the epithelial and mesenchymal scores for cells sampled from intestine, liver, lung, and skin. Organs are indicated by colors, and cell types are indicated by shapes. The *x*-axis represents the epithelial score, and the *y*-axis represents the mesenchymal score. **b** Scatterplots showing the changes in epithelial, mesenchymal, and stemness scores for epithelial cells sampled from intestine, liver, lung, and skin during development, as inferred by PC1 in Fig. 3a. The Pearson correlation coefficient between each score and PC1 is calculated

We defined an epithelial score (E-score) by averaging the expression of E-cadherin, ZO-1, claudins, occludin, cytokeratins, and type IV collagen, and a mesenchymal score (M-score) by averaging the expression of vimentin, FSP-1, α-SMA, fibronectin, N-cadherin, and type I and type III collagen (see Additional file 2 for gene list). Interestingly, epithelial cells of different organs showed different E/M characteristics during this developmental stage, though they were all composed of epithelial parenchyma (Fig. 6a). Liver possessed the lowest E-score and a moderate M-score, while lung had the lowest M-score and a moderate E-score. The E- and M-scores of intestine and skin seemed more scattered. A previous study proposed that as the mesenchymal phenotype increases, stemness is acquired during EMT [25, 34]. Thus, we also defined a stemness score (S-score) by averaging all the genes of the Gene Ontology (GO) term “stem cell population maintenance” (GO: 0019827). When we ordered cells along the pseudodevelopmental timeline as inferred from the PC1 axis in Fig. 3a, we did see clear patterns (Fig. 6b). Throughout the process of development, all three scores of epithelial cells in the intestine and liver decreased, exhibiting a negative correlation with the PC1 axis. This result was consistent with the above finding that early epithelial cells (cluster 1) in these two organs shared the most DEGs (Fig. 3c), which were enriched in terms related to stemness and mesenchymal features, such as signaling pathways regulating pluripotency of stem cells, osteoblast differentiation, and extracellular matrix organization. However, all three scores for lung increased during development and showed a positive correlation with the PC1 axis. This might be because organogenesis for lung begins during this period. Since epithelial cells in lung at E18 barely expressed genes underlying mesenchymal features such as *Vim*, *Fn1*, *Cdh2*, and *Co13a1*, we expected that these three scores would decrease later during development (Fig. 5b). We also reasoned that the intestine and liver would first show increases in all these three scores before E9.5 and then decreases, which was what we detected during E9.5–E11.5. Although the E- and M-scores during development for the skin epithelial cells did not change as in the other three organs, the S-score did decrease during their development. These results all show the remarkable plasticity of epithelial cells during organogenesis. To conclude, this E/M hybrid state seemed to be a common process in endodermal organs with epithelial parenchyma, and the E/M hybrid state had a positive correlation with stemness in intestine, liver, and lung during organogenesis.

**Fig. 7:**
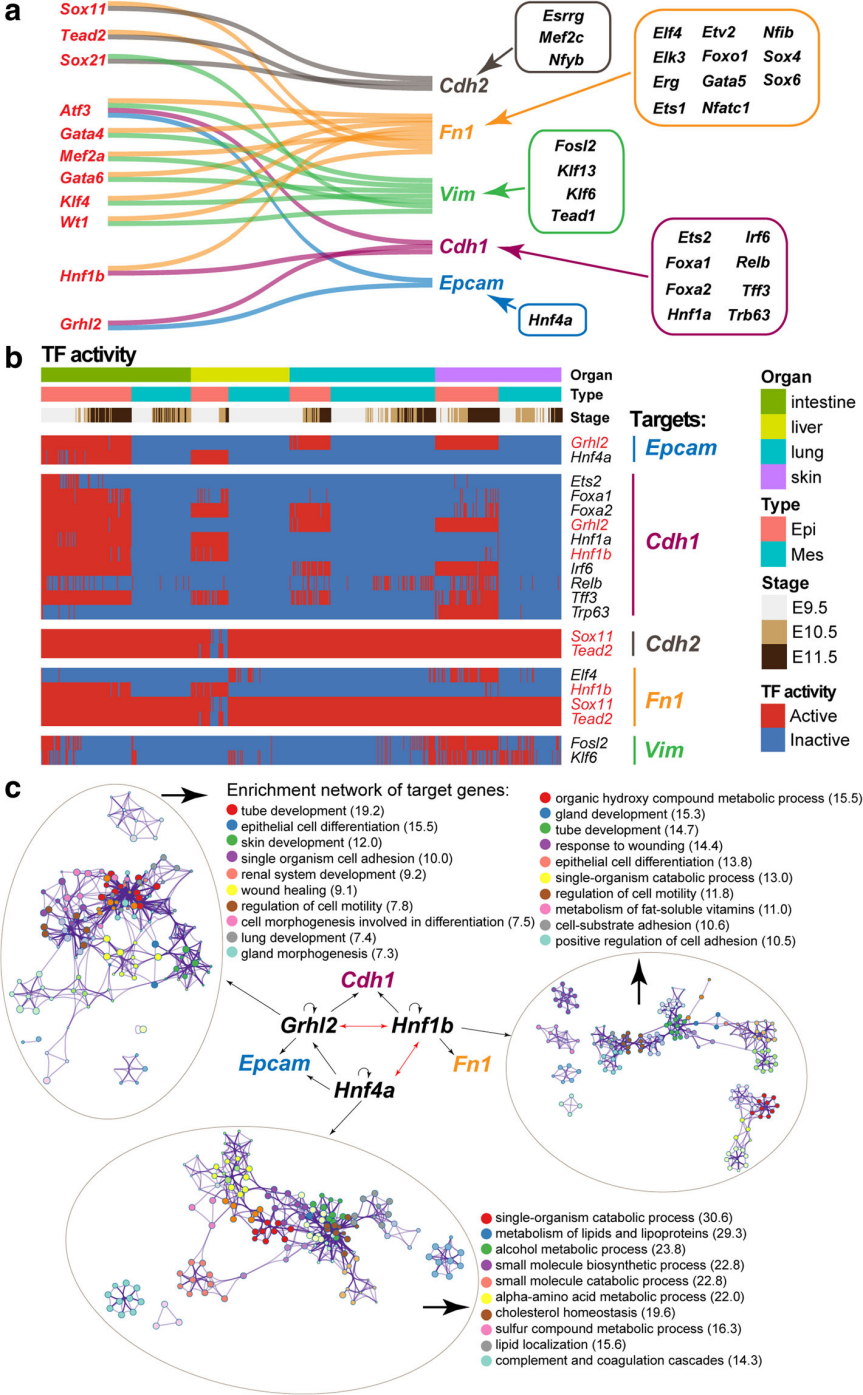
Transcription factors (*TFs*) regulating key epithelial and mesenchymal markers. **a** TFs that positively regulate key epithelial and mesenchymal markers. TFs on the *left* can regulate more than one marker, and marker-specific TFs are shown on the *right*. TFs and their targets are linked by lines. **b** Heatmap showing the TF activity in epithelial and mesenchymal cells sampled from intestine, liver, lung, and skin. The color key from *blue* to *red* indicates low to high TF activity, respectively. **c** Enrichment networks for target genes of *Grhl2*, *Hnf1b*, and *Hnf4a*. Their interactions are also indicated by *arrows*

Next, we utilized the TF regulon activity obtained by SCENIC to detect what TFs regulated these key epithelial (*Epcam* and *Cdh1*) and mesenchymal markers (*Vim*, *Fn1*, and *Cdh2*) in the four organs with epithelial parenchyma. Two criteria were used to identify the TFs: first, we only kept co-expressed TFs with positive correlations, i.e., potential activation associations; second, we only kept TFs whose binding motif was over-represented in the search space around the transcription start site (TSS) of genes. As shown in Fig. 7a, these five markers all had their specific TFs. In addition, several TFs could regulate more than one marker. We further checked their TF activity across the four organs and pruned TFs with low activity. The remaining TFs are shown in Fig. 7b. We only detected *Grhl2* and *Hnf4a* as responsible for the expression of *Epcam* in epithelial cells, and these two TFs had different roles. *Grhl2* regulated *Epcam* and *Cdh1* and was active in epithelial cells of the intestine, lung, and skin but not the liver. The expression of *Epcam* in the liver was inferred to be regulated by *Hnf4a*, which was also active in the intestine. *Hnf1b* regulated both *Cdh1* and *Fn1*, and it was limited to epithelial cells of the intestine and liver. Epithelial cells of the liver seemed quite different from the other three organs in terms of TF activity. Meanwhile, the TFs that were active in the liver were all shared with the intestine. This might be one of the reasons why the intestine and liver possessed similar E- and M-score patterns during development as shown in Fig. 6b. For *Cdh2*, *Fn1*, and *Vim*, we did not identify epithelial cell-specific TFs, except *Hnf1b*, which was shared by the intestine and liver.

We selected three epithelial cell-specific TFs, *Grhl2*, *Hnf4a*, and *Hnf1b*, to explore their targets because only *Grhl2* and *Hnf4a* were identified to activate the important epithelial gene *Epcam*, and *Hnf1b* regulated both *Cdh1* and *Fn1* (Fig. 7b and c). These three TFs were all self-regulating and had strong interactions with each other. Targets of *Grhl2* and *Hnf1b* shared several enriched terms, including tube development, epithelial cell differentiation, and regulation of cell motility. Targets of *Hnf1b* were also enriched in terms related to cell adhesion, such as cell-matrix adhesion, which was related to mesenchymal features. In addition, metabolism-related terms were enriched among the targets of *Hnf4a*.

### Development of hematopoietic cells

**Fig. 8:**
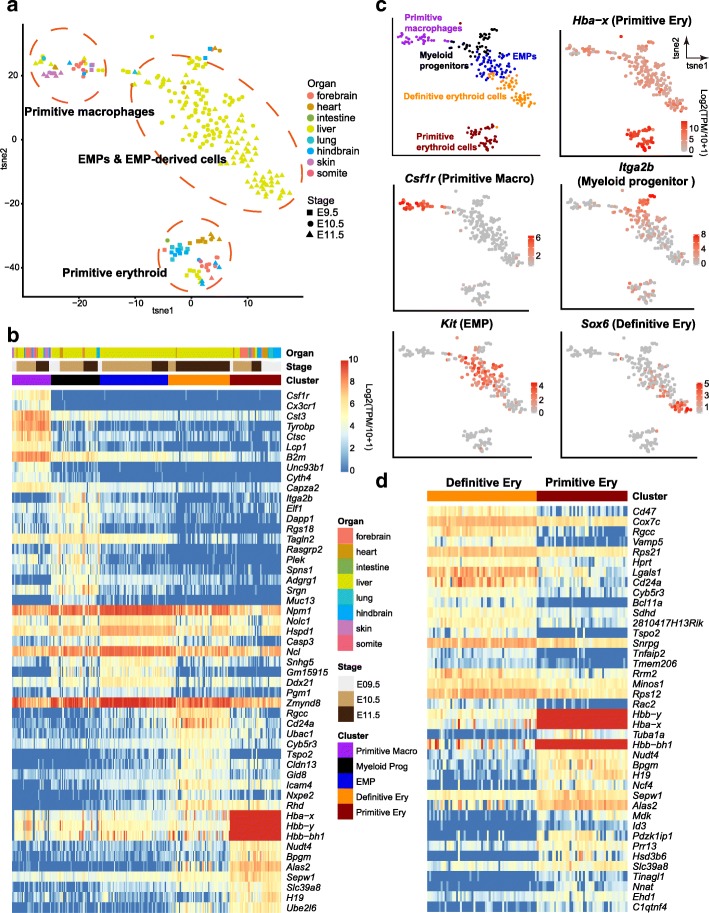
Expression patterns of hematopoietic cells. **a** Expression-based t-SNE plot of hematopoietic cells. Cells sampled from different organs are indicated by colors, and their developmental stages are indicated by shapes. **b** Heatmaps showing the top 10 DEGs of each cluster. The color key from *blue* to *red* indicates low to high gene expression, respectively. **c** The expression of representative markers mapped on the t-SNE plot in **a**. **d** Heatmaps showing the top 20 DEGs between definitive primitive erythroid cells

Blood cell synthesis already started to work at E7.25, before the emergence of hematopoietic stem cells (HSCs), and this HSC-independent hematopoiesis was necessary to sustain normal development during mouse early embryogenesis [35]. This HSC-independent hematopoiesis included two partially overlapping waves of progenitors, the primitive and definitive progenitors. The definitive progenitors seeded the fetal liver to initiate hematopoiesis, and in our data, a large proportion of cells sampled from the liver belonged to these populations. We captured these two waves of hematopoietic cells, which were further divided into five clusters (Fig. 8a and b). The expression patterns of DEGs confirmed the classification accuracy. Based on known markers, we assigned these clusters as a primitive macrophage cluster, as they expressed *Csf1r* and *Cx3cr1*, and the majority of cells were not from liver; a myeloid progenitor cluster, expressing *Itga2b* and *Adgrg1*; an erythro-myeloid progenitor (EMP) cluster expressing *Kit*; a definitive erythroid cluster (*Sox6* and *Bcl11a*); and a primitive erythroid cluster (*Hba-x*) (Fig. 8b and c). EMPs exhibited clear bi-potential differentiation ability, as shown in the t-SNE plot (Fig. 8c).

Obtaining both primitive and definitive erythroid cells provided a great opportunity to make comparisons between them (Fig. 8d). During this stage, primitive erythroid cells had already taken part in the blood circulation, while definitive erythroid cells were just differentiated from EMPs and experienced expansion. We also identified several surface markers and TFs for further studies of these cell types (Additional file 1: Figure S4a). All the identified surface markers were highly expressed in definitive erythroid cells. Also, both definitive and primitive erythroid cells had their specifically expressed TFs, for example, *Bcl11a*, *Hmgb3*, *E2f4*, *Tfdp1*, and *Sox6* for definitive erythroid cells, and *Id3*, *Arid3a*, *Lmx1a*, *Tcf7l2*, and *Sox11* for primitive erythroid cells. In particular, *Sox6* and *Lmx1a* were exclusively expressed in definitive and primitive erythroid cells, respectively, even compared with other hematopoietic cells. *Sox6* was important for the definitive erythroid maturation and suppressed the expression of embryonic globin genes, while *Lmx1a* positively regulated the transcription of the insulin genes. We also performed quantitative polymerase chain reaction (qPCR) for five representative markers (*Alas2*, *Slc4a1*, *Bcl11a1*, *Cd47*, *Cd24a*) to valid our identification. The results were consistent with those of the single cell RNA-seq (Additional file 1: Figure S4c).

### Development of neuronal cells

**Fig. 9:**
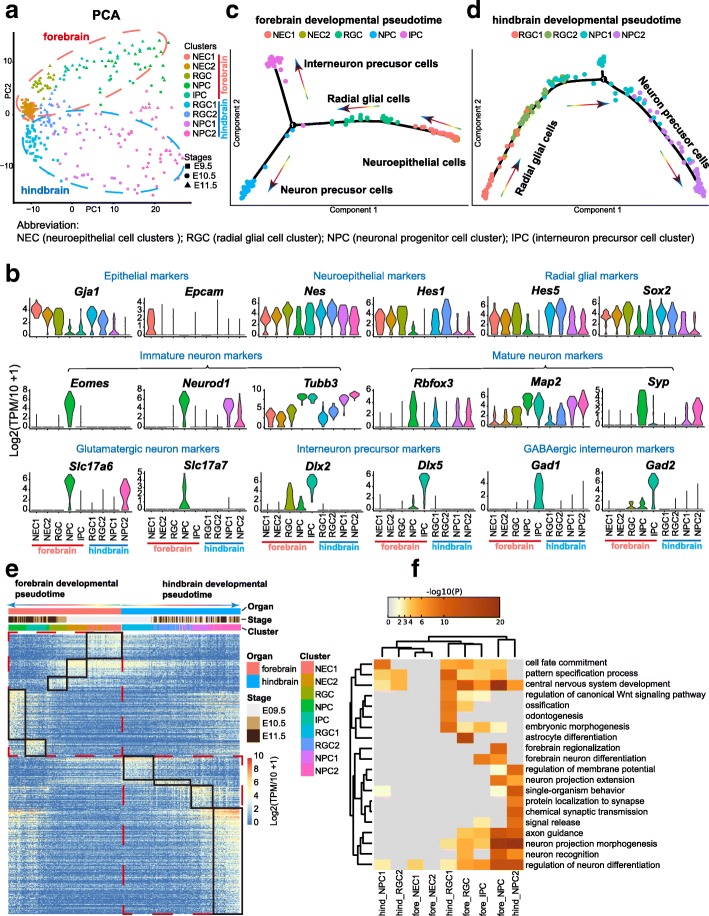
Expression patterns of neuronal cells. **a** PCA plot showing the neuronal cells sampled from the forebrain and hindbrain. Cells from different clusters are indicated by colors, and their developmental stages are indicated by shapes. Abbreviation information is shown on the bottom. **b** Violin plots showing the expression of representative markers in each cluster. **c** Developmental pseudotime of forebrain cells inferred by Monocle2. Clusters are indicated by colors. **d** Developmental pseudotime of hindbrain cells inferred by Monocle2. Clusters are indicated by colors. **e** Heatmaps showing the DEGs of each cluster compared with all other clusters. Cells are arranged by the developmental pseudotime from **c** and **d**. **f** Heatmaps showing enrichment of DEGs of each cluster. The color key from *gray* to *brown* indicates high to low *P* values, respectively

We next analyzed neuronal cell development in the forebrain and hindbrain. By combining the regulon matrix and the expression matrix, forebrain and hindbrain neuronal cells were further divided into five and four clusters, respectively. Established markers and enriched terms let us assign them into appropriate identities (Fig. 9a, b, and e; Additional file 1: Figure S5a): for the forebrain, two neuroepithelial cell clusters (NECs), one radial glial cell cluster (RGC), one neuronal progenitor cell cluster (NPC), and one interneuron precursor cell cluster (IPC); and for the hindbrain, two RGCs and two NPCs. Cells were substantially stretched across the PCA plot (Fig. 9a). The first axis of the PCA explained the development of neuronal cells in two organs, ordering cells during maturation or differentiation, suggesting a largely common developmental route in these two sources of neuronal cells. Thus, we reasoned that the gene expression underlying developmental variation was shared by the two organs. The DEGs ordered by developmental pseudotime also supported this opinion: genes specifically expressed in both early and late developmental stages were largely shared by the two organs, and so were the enriched terms (Fig. 9c, d, e, and f; Additional file 1: Figure S5a). Neuronal cells of the forebrain and hindbrain at early stages both expressed *Id2*, *Id3*, *Hes1*, *Nes*, *Vim*, and *Sox2*, which were important for the differentiation of neuronal cells, while at later stages they both expressed *Stmn2* and *Stmn3*, which encoded proteins regulating neuronal growth, as well as several neuron markers, such as *Tubb3* and *Map2* (Fig. 9b; Additional file 1: Figure S5a).

However, the differences between forebrain and hindbrain were also clear. First, the cell type composition was quite different. At the later stage, IPCs expressing *Dlx2*, *Dlx5*, *Gad1*, and *Gad2* emerged in the forebrain but not in the hindbrain (Fig. 9b). Second, the developmental processes of the two organs were asynchronous. Even at E9.5, all four cell types were detected in the hindbrain, while this pattern was not seen in the forebrain. Instead, the majority of cells were NECs or RGCs, and specific cell types were limited to a certain timeline in the forebrain, with more mature cells only found at later stages (Fig. 9a). However, it was interesting that, at later stages, neuronal cells of the forebrain seemed to be more mature than those of the hindbrain, as evidenced by the expression of the mature neuron markers *Syp*, *Map2*, and *Rbfox3* and the existence of interneuron cells (Fig. 9b). Third, the second axis of PCA separated forebrain and hindbrain, implying the existence of organ-specific gene expression patterns between the two organs (Fig. 9a). Indeed, several such genes were identified, for example, *Foxg1* and *Emx2* for forebrain, which are related to forebrain development and regionalization, and *En1*, *Wls*, and *Rfx4* for hindbrain, which are related to hindbrain development and regionalization (Additional file 1: Figure S5b and S5c).

### Development of mesoderm organs (heart and somite)

Among the mesoderm-derived organs, we captured the heart and the somites. Cells from the heart were classified into six clusters within three cell types through the regulon matrix: two cardiomyocyte clusters (CMs) on the basis of high expression of *Ttn* and *Myl1*; two endothelial cell clusters (EDs) specifically expressing *Eng* and *Pecam1*; and two epicardial cell clusters (EPs), which specifically expressed *Upk1b*, *Upk3b*, and *Wt1* (Additional file 1: Figure S6a). As expected, the two clusters within each of the three cell types displayed a pattern of continuous development. Cells in earlier clusters mainly consisted of cells from E9.5 embryos, whereas cells in later clusters mainly consisted of cells from E10.5 and E11.5 embryos. We also compared the two clusters within each cell type (Additional file 1: Figure S6b). Of the two CMs, the early one highly expressed *Sdf2l1*, *Creld2*, *Erp44*, and *Tmem41b*, while the later one highly expressed *Rps20*, *Pf4*, *Gm12657*, and *Pln*. In the two EPs, genes such as *Ewsr1*, *Pkm*, *Lsp1*, and *Dkc1* were highly expressed in the early one, while genes such as *Clca3a1*, *Hpgd*, *Aldh1a2*, and *S100a10* were highly expressed in the later one.

**Fig. 10:**
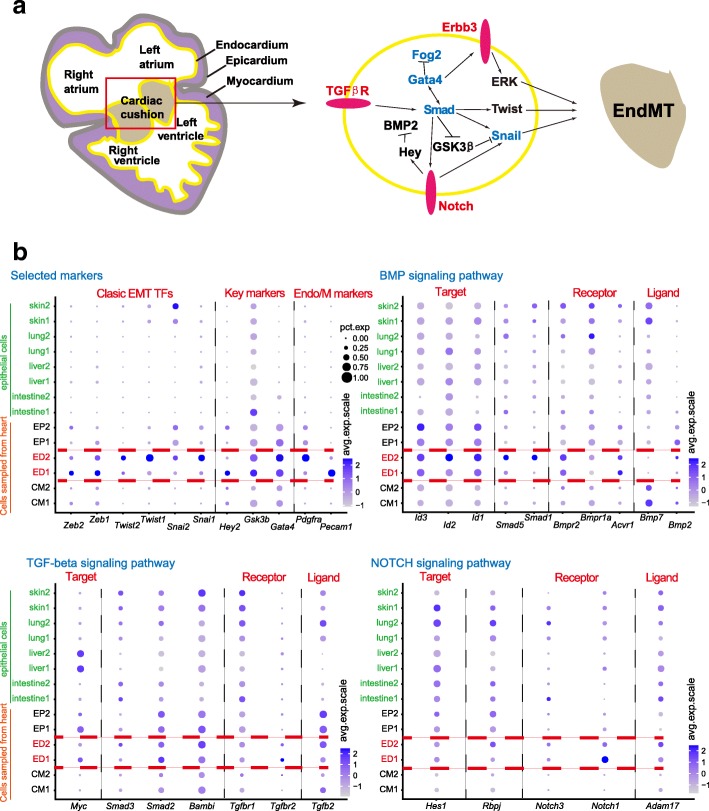
Expression patterns of representative markers during endothelial-to-mesenchymal transition (*EndMT*) in heart. **a** Schematic of EndMT and the key markers and signaling pathways modified from Lim and Thiery [5]. **b** The expression of key markers and signaling pathways during EndMT in heart. The epithelial clusters of intestine, liver, lung, and skin are also shown. The color key from *gray* to *purple* indicates low to high average gene expression, respectively. The *dot size* indicates percentage of cells expressing a certain marker

In addition, we captured the endothelial-mesenchymal transition (End-MT) mainly in the ED2 cluster, which led to the formation of the cardiac cushion (Fig. 10a and b). We thus made comparison between End-MT and the E/M hybrid state detected above. As shown in Fig. 10b, endothelial cell clusters ED1 and ED2 expressed the classical EMT-inducing TF markers (*Snai1/2*, *Zeb1/2* and *Twist1/2*). However, the expression patterns were different: *Zeb1/2* expression was high in ED1, while *Snai1/2* and *Twist1/2* expression was high in ED2, suggesting distinct roles of these TFs in Endo-MT. With the upregulation of *Snai1/2* and *Twist1/2* and downregulation of *Zeb1/2*, the endothelial cells gradually lost endothelial markers (e.g., *Pecam1*) and gained mesenchymal markers (e.g., *Pdgfra*). In contrast, we did not observe expression of these genes in epithelial cells with the E/M hybrid state.

Several pathways participated in Endo-MT. For example, cardiomyocytes secreted bone morphogenetic protein (BMP) ligands to interact with the receptors on the target cell surface to activate downstream genes through Smad proteins; transforming growth factor beta (TGFβ) and Notch signaling pathways were also activated to promote Endo-MT. Transcription factors such as *Hey2* and *Gata4* were also important for this process. Again, no clear pattern was seen in epithelial cells with E/M hybrid state.

Mesoderm-derived cells collected from the somites were divided into five further clusters (Additional file 1: Figure S7a and S7b). They developed from cells with a dermomyotome feature (cluster 1) in two directions as revealed by pseudotime analysis in Monocle2 (Additional file 1: Figure S7a). One was myotome muscle cells (cluster 3), and the other was mesenchymal sclerotomes (cluster 5). The first cluster consisted of cells from E9.5 somites with dermomyotome features, expressing high levels of *Prtg*. During mouse somitogenesis, *Prtg* is restricted to the dorsal parts of the spinal cord (the roof plate and neighboring cells) and of the somite (the dermomyotome). Cluster 2 was composed of skeletal muscle cells because of its specific expression of *Fos* and *Erg1*, which are related to skeletal muscle cell differentiation. Cluster 3 included a population of muscle cells derived from the myotome that highly expressed *Tpm1* and *Tpm2*. Cluster 4 showed features of a transitional state. Cells in cluster 5 were presumably from the mesenchymal sclerotome, as they expressed the sclerotome marker *Pax1*, the mesenchymal gene *Col3a1*, and a series of Hox genes, such as *Hoxd11* and *Hoxa11os* (Additional file 1: Figure S7b).

In addition, 10 somite cells were grouped into the neuronal cell group. We thus compared these neuronal cells sampled from the somites with the neuronal cells from the brain and the somite mesoderm-derived cells (Additional file 1: Figure S7c, S7d, and S7e). We noticed that *Neurod4* was highly expressed in the somite neuronal cells compared with the other two groups. *Neurod4* is a member of the neurogenic differentiation factor family and is involved in the Ngn2-regulated neuronal differentiation pathway, which coordinates the onset of cortical gene transcription. We used the Meta-analysis workflow in Metascape to combine these two DEG sets of the somite neuronal cells for comparison with the other two groups. The shared enriched terms included several neuronal development-related processes, the Notch signaling pathway, and cell fate commitment.

## Discussion

In this study, we systematically analyzed the transcriptomic features in the major organs of E9.5 to E11.5 mouse embryos at single-cell resolution. The gene and transcript coverages make our data more sensitive than those at 10X or Drop-Seq’s level, which makes our data more accurate and comprehensive (Additional file 1: Figure S1a). The developmental characteristics revealed in various organs and cell types broaden our horizons about mouse organogenesis and facilitate further deeper functional studies on mammalian embryonic development.

We highlight the importance of regulon activity-based methods to reveal evolutionary or developmental relatedness across multiple organs (Fig. 1a and b). As mentioned in several studies, the expression patterns of realizer genes tend to reflect functional similarities, while regulatory genes are more likely to assess the function of cells [19–22]. However, regulatory gene-based methods also have shortcomings, for example, the limited resolution. It is difficult to detect weak developmental differences at a fine scale. Thus, in this study we first used a regulon matrix constructed by SCENIC to perform the hierarchy clustering and then combined the results with results from the expression matrix to reveal subtle developmental relationships. Based on the regulon activity, we obtained four major groups with epithelial, mesodermal, hematopoietic, and neuronal features (Fig. 1a and b). Subsequently, we comprehensively studied the transcriptomic and developmental features within each of them.

We first focused on the development of epithelial cells sampled from intestine, liver, lung, and skin. However, due to the important role of mesenchymal cells in inducing and specifying epithelial identity [1], it is necessary to take mesenchymal cells into account when studying the development of organs composed of epithelial parenchyma (Fig. 2a and b). Although several studies based on bulk RNA-seq have already tried to identify the developmental expression pattern of these organs [9–11], they could hardly consider the mesenchymal effects. Even worse, bulk data would lead to a mixed result of both epithelial and mesenchymal cells, rather than the pure development of a certain organ. Thus, our data reveal an accurate and pure expression pattern in each of these organs throughout development (Fig. 2a). In addition to the unique expression pattern of these two cell types within each organ, we identified the interactions between epithelial and mesenchymal cells, which guarantee the normal development of a certain organ (Fig. 2b and c). The developmental comparison between these organs also provides a valuable resource for others to study organ specification (Fig. 3).

Another noteworthy finding is the prevalent E/M hybrid state in epithelial cells. As shown in Fig. 4a, nearly all epithelial cells during this stage co-expressed the epithelial (*Epcam*, *Cdh1*, *Cldn6*, *Krt18*, etc.) and mesenchymal (*Vim*, *Fn1*, *Sparc*, etc.) markers, exhibiting the E/M hybrid phenomenon. The immunostaining results also unambiguously validate this (Fig. 4b; Additional file 1: Figure S3a). However, this phenomenon was not the same as classical EMT events, in which typical TFs (*Zeb1/2*, *Twist1/2*, *Snai1/2*, etc.) play crucial roles. These TFs were barely expressed in the epithelial cells during this developmental period (Fig. 5a). Additionally, this E/M hybrid state was different from End-MT in heart endothelial cells, as shown in Fig. 10b. In addition, adult or mature epithelial cells did not show this E/M hybrid state, as revealed in adult intestine cells, adult liver cells, and E18 lung cells (Figs. 4c and 5b). Therefore, this E/M hybrid state seems to be an important feature of epithelial cells during organogenesis and may play a crucial role in epithelial development, such as making them move or migrate collectively, while keeping their epithelial feature.

Surprisingly, this E/M hybrid state tended to be a common process across the endodermal organs with the epithelial parenchyma, because the epithelial cells of intestine and liver exhibit the pattern of E- and M-scores gradually decreasing during E9.5–E11.5 (Fig. 6b). The epithelial cells of lung showed an opposite trend: E- and M-scores gradually increased during this period. We would expect a similar E- and M-score pattern as in the intestine and liver to occur later, given that the lung organogenesis is in general later than the intestine and liver organogenesis and that at E18 the E/M hybrid state is no longer seen in the epithelial cells of lung (Fig. 5b). However, the E- and M-scores of the skin epithelial cells exhibited no correlation during E9.5–E11.5. Since the skin is derived from the ectoderm, whether this could reflect the differences between the ectodermal and endodermal epithelial cells in terms of E/M features requires further study.

Such an E/M hybrid state has also been reported in tumorigenesis and circulating tumor cells (CTCs) [36]. We thus downloaded two single-cell RNA-seq datasets of cancer [32, 33] to check whether this phenomenon was also present in these datasets. Two carcinoma datasets (breast and lung cancer) also displayed a universal E/M hybrid state with the co-expression of *EPCAM* and *VIM*. However, there also existed several differences in terms of the expression pattern of classical EMT-inducing TFs (*Zeb1/2*, *Twist1/2*, and *Snai1/2*): the hybrid state in lung cancer cells seemed to be more dependent on these TFs in comparison with that in breast cancer. Considering that these lung cancer cells were patient-derived xenograft (PDX) tumor cells sampled from a lung adenocarcinoma patient tumor xenograft, other human in vivo lung cancer data should be checked for comparison.

A possible explanation for this E/M hybrid state is that the E/M hybrid state lets cells acquire stemness and more invasive features, as shown by several studies [25]. Indeed, we did see that the stemness score had a positive correlation with the E- and M-scores (Fig. 6b). During the process of development, these three scores all decreased in epithelial cells of the intestine and liver, while they increased in the lung. Whether the similar E/M hybrid pattern in developing epithelial organs and carcinomas is regulated by similar mechanisms needs further investigation. If the hypothesis we put forth above is true, that carcinomas utilize the embryonic mechanism to perform metastasis, it will be helpful for researchers in studying cancer metastasis. They can simply examine what happens to these organs during organogenesis.

The regulatory mechanisms underlying this E/M hybrid state are nonlinear, and they may involve different levels, such as transcriptional control, epigenetic modifications, and alternative splicing [7]. We still think our data can provide valuable insights into the hybrid state. We identified several TFs that potentially regulate key epithelial (*Epcam* and *Cdh1*) and mesenchymal (*Vim*, *Fn1*, and *Cdh2*) markers in the four analyzed organs with epithelial parenchyma. As shown in Fig. 7b, these five markers were all regulated by several TFs. Among these TFs, *Grhl2* and *Hnf4a* were responsible for activating *Epcam*. However, they had different roles: *Grhl2* regulated *Epcam* expression in epithelial cells of intestine, lung, and skin, while *Hnf4a* regulated *Epcam* expression in intestine and liver. *Grhl2* is a key regulator of the E/M hybrid state of lung cancer cells [28], and *Hnf4a* is the master effector of mesenchymal-epithelial transition (MET) and is able to maintain hepatocyte identity [37]. Another noteworthy TF is *Hnf1b*, which is active in epithelial cells of intestine and liver and regulated both *Cdh1* and *Fn1* (Fig. 7b). *Hnf1b* is a novel oncogene that is able to induce cancerous phenotypes, EMT, and invasive phenotypes [38]. These three TFs were all self-regulating and interacted with each other (Fig. 7c).

Additionally, we captured two waves of HSC-independent hematopoiesis. Our data show that the two partially overlapping waves of progenitors, the primitive and definitive progenitors, were unambiguously discriminated by single-cell RNA-seq analysis. The differentiation of EMPs was also observed in this study. Furthermore, by comparing primitive and definitive erythroid cells, we identified several candidate surface markers and TFs, which will be of great help for future functional studies of the development of embryonic erythroid cells. We also deeply explored the different developmental patterns between the forebrain and hindbrain. The developmental processes of both forebrain and hindbrain were characterized. However, the forebrain and hindbrain neuronal cells were quite different in terms of cell types, developmental routes, and expression patterns, indicating temporal and spatial heterogeneity in the embryonic brain. From heart, we captured the End-MT process in the endothelial cells, which was quite different from the E/M hybrid state identified above. End-MT depended on the classical EMT-inducing TF markers (*Snai1/2*, *Zeb1/2*, and *Twist1/2*) (Fig. 10b). In addition, from the somites, we obtained several rare neuronal cells that were different from the brain neuronal cells or other somite cells.

## Conclusions

In summary, our study elucidates the transcriptome landscape of mouse organogenesis from E9.5 to E11.5 at single-cell resolution. We reveal many detailed biological features of the major mouse organs, such as the developmental features of each cell type and the expression patterns of critical genes. Our data should be useful in future functional studies of lineage formation and for obtaining further insights into the molecular mechanisms of mouse organogenesis.

## Methods

### Mouse embryo dissection and single-cell isolation

Cells were separated from the tissues and organs of E9.5–E11.5 C57BL/6 J mouse embryos. Briefly, E9.5–E11.5 mouse embryos were obtained by euthanizing pregnant mice and transferring the embryos to a petri dish containing fresh, sterile Dulbecco’s phosphate-buffered saline (DPBS). The embryos were washed extensively to remove any maternal contaminants and excess blood. Then, the embryos were placed in Dulbecco’s modified Eagle’s medium (DMEM) containing 10% fetal bovine serum (FBS). The tissues and organs used in this study included the forebrain, hindbrain, skin, heart, liver, intestine, lung, and somites, which were carefully separated with microdissecting forceps under a dissection microscope. These organs were digested with 0.05% trypsin-ethylenediaminetetraacetic acid (EDTA) at 37 °C for approximately 5 min, and then DMEM (containing 10% FBS) was added to stop the digestion. The cells were then further dissociated into single-cell suspensions by gentle pipetting with a mouth pipette.

### Single-cell cDNA amplification and library construction

Single-cell cDNA amplification was carried out by using the STRT protocol with several modifications to allow for multiplexed single-cell RNA-seq. Briefly, after trypsinization of each dissected organ and tissue to obtain the single cells, a mouth pipette was used to pick single cells into 2 μL of cell lysis buffer in 200-μL PCR tubes, each containing 0.1 U/μL RNase Inhibitor (Takara, 2313B), 0.0475% Triton X-100 (Sigma-Aldrich, X100), 2.5 μM deoxynucleotide triphosphate (dNTP) mixture (Thermo, R0193) and 2.5 μM barcode-reverse transcriptase (RT) primers (TCAGACGTGTGCTCTTCCGATCT-XXXXXXXX-NNNNNNNN-T25, where X represents the nucleotides of the designed cell-specific barcodes and N represents the unique molecular identifier [UMI], see Additional file 2). To lyse the cells, the tube was first vortexed thoroughly and placed in a thermocycler at 72 °C for 3 min to release the linearized RNA molecules. Then, the reaction was immediately quenched on ice. After the reaction was centrifuged, 2.85 μL of RT mixture (40 U SuperScript II reverse transcriptase [Invitrogen, 18,064,071], 5 U RNase Inhibitor, 5× Superscript II first-strand buffer, 25 mM dithiothreitol [DTT], 5 M betaine [Sigma-Aldrich, B0300], 30 mM MgCl_2_ [Sigma-Aldrich, 63,020], and 1.75 μM template switch oligo [TSO] primer [AAGCAGTGGTATCAACGCAGAGTACATrGrG+G, where rG represents riboguanosine one (+G), and +G indicates the LNA-modified guanosine]) were added into the single-cell lysate. Reverse transcription was carried out in the thermocycler at 25 °C for 5 min, 42 °C for 60 min, 50 °C for 30 min, and then 70 °C for 10 min.

After reverse transcription, 7.5 μL of PCR mixture (6.25 μL 2× KAPA HiFi HotStart ReadyMix [KK2602], 300 nM ISPCR oligo [AAGCAGTGGTATCAACGCAGAGT], and 1 μM 3′ Anchored oligo [GTGACTGGAGTTCAGACGTGTGCTCTTCCGATC]) were added to each reaction. The sample was amplified with four cycles of 98 °C for 20 s, 65 °C for 30 s, and 72 °C for 5 min, followed by 10–15 cycles of 98 °C for 20 s, 67 °C for 15 s, and 72 °C for 5 min; and finally 72 °C for 5 min.

The amplified samples with different barcodes (up to 96 barcodes) were pooled together. The pooled cDNAs were first purified with DNA Clean & Concentrator-5 (DC2005; Vistech, Beijing, China) and eluted in 50 μL of H_2_O. After the sample was further purified twice with 0.8× Ampure XP beads (Beckman, A63882), the cDNAs were used for a second round of amplification with biotinylated index primer (/Biotin/CAAGCAGAAGACGGCATACGAGATindexGTGACTGGAGTTCAGACGTGTGCTCTTCCGATC) and ISPCR oligo for an additional four to five cycles. Subsequently, the biotinylated cDNAs were again purified with 0.8× Ampure XP beads. Next, the cDNAs were sheared into approximately 300-bp fragments with a Covaris sonicator. The fragmented cDNAs containing barcode and UMI sequences were then enriched by using Dynabeads® MyOne™ Streptavidin C1 (Invitrogen, 65,002).

Libraries were prepared using KAPA Hyper Prep Kits (KK8505). The NEB U-shaped adapter was used for ligation. Finally, the adapter-ligated fragments were amplified by using an Illumina QP2 primer (CAAGCAGAAGACGGCATACGA) and a short universal primer (AATGATACGGCGACCACCGAGATCTACACTCTTTCCCTACACGAC) for 8–10 cycles. The libraries were sequenced on an Illumina Hiseq4000 platform to generate 150-bp paired-end reads (sequenced by Novogene).

### Processing of single-cell RNA-seq data

We first segregated raw reads on the basis of the information from the cell-specific barcodes in read 2 of the paired-end reads. Then, the TSO sequence and polyA tail sequence in read 1 were trimmed with customized scripts. Subsequently, sequences in read 1 that had low-quality bases (*N* > 10%) or that were contaminated with adapters were discarded. The stripped read 1 sequences were then aligned to the mm10 mouse reference genome (University of California, Santa Cruz, UCSC) using TopHat (version 2.0.12) [39]. We used htseq-count from the HTSeq package [40] to count uniquely mapped reads, which were then grouped on the basis of the cell-specific barcodes. For each gene, duplicated transcripts with identical UMIs were removed. Finally, for each gene in each cell, the copy number of the transcript was quantified on the basis of the number of distinct UMIs of that gene.

In total, we sequenced 1916 single cells (see Additional file 3). Cells with fewer than 2000 detected genes were removed, leaving 1819 cells for downstream analysis. Because most of our single cells did not reach one million UMIs, we used log_2_(TPM/10 + 1) rather than log_2_(TPM + 1), where TPM refers to transcripts per million, to normalize the expression levels. This procedure prevented each transcript from being counted multiple times.

### Saturation analysis of sequenced data

To make sure the sequencing depth of our dataset was sufficient, we randomly selected one set of sequencing data (which included 64 cells sampled from the E11.5 intestine) to down-sample the raw sequencing data to 10, 20, 30, 40, 50, 60, 70, 80, and 90% of its original data. Next, we obtained the numbers of detected genes in each of these down-sampling datasets and compared them with the numbers of detected genes in the original dataset. The results are shown in the boxplot in Additional file 1: Figure S1b.

### Nonlinear dimensional reduction (t-SNE) and clustering based on the regulon matrix and the expression matrix

To explore the evolutionary or developmental relationships among organs, we performed unsupervised clustering analysis adjusted by the random forest algorithm using a binary regulon matrix and a gene expression matrix. The regulon matrix was generated by SCENIC [23] using UMI counts. SCENIC is an algorithm that can reconstruct gene-regulatory networks (GRNs) and identify stable cell states from single-cell RNA-seq data. For details, access the website of SCENIC: http://aertslab.org/#scenic. In brief, SCENIC first inferred co-expression modules, which were subsequently trimmed by *cis*-regulatory motif analyses, leaving a subset of pruned modules termed regulons. SCENIC then scored all cells for the activity of each regulon by calculating the enrichment of the regulon as an area under the recovery curve (AUC) across the ranking of all genes in a particular cell, whereby genes were ranked by their expression values. Finally, a binary regulon activity matrix was obtained, and using this matrix, we carried out nonlinear dimensional reduction (t-SNE) through the Rtsne package in R.

For the expression matrix, we analyzed our 1819 single-cell data in the form of log_2_(TPM/10 + 1) expression values using the Seurat method [41] (for details, see http://satijalab.org/seurat/). Specifically, genes were considered expressed only if their expression level was ≥ 1. Genes expressed in < 3 cells and cells with ≤ 2000 detected genes were discarded. Highly variable genes with average expression > 1 and dispersion > 1 were used as inputs for t-SNE analysis.

The clustering method was modified from Lake et al. [42], and the scripts were also attached in the supplemental files of that paper. This method combined unsupervised clustering to reveal heterogeneity in cell subtypes and supervised classification to fine-tune clusters. Each time, two clusters were obtained through this method. In brief, (1) we first performed hierarchical clustering using Pearson correlation distance metrics and obtained the first two split clusters. If the input was the expression matrix, the highly variable genes were first identified and then we performed hierarchical clustering. (2) We then used a 10-fold random forest feature selection to choose feature genes dividing the two clusters. (3) Samples with internal vote probabilities > 0.6 were selected for each class as the training set to achieve an optimal classifier, which was used to predict the rest of the samples. (4) We performed 100 runs of 10-fold random forest cross-validation (CV) and discarded samples with internal vote probabilities < 0.55. We used internal vote probabilities > 0.55 (higher than default = 0.5) as the cutoff to reduce the ambiguity of sample voting. (5) To obtain more finely tuned clusters, steps 1–4 were recursively repeated on the newly formed classes. We applied the classification method to both the regulon matrix and the expression matrix. The hierarchy trees in Fig. 1c and Additional file 1: Figure S1c were constructed by the order of obtained clusters through this method.

### Identification of top TFs, DEGs, and GO terms

To identify top-ranking TFs for a certain cluster, we averaged the TFs of the binary regulon matrix for this cluster and the rest. The ranking was set by the difference between the average value of this cluster and the average value of the rest. A bigger difference corresponded to a higher ranking. For DEGs, analysis was carried out in Seurat. We used the Seurat function find_all_markers (thresh.test = 1, test.use = “roc”) to identify unique cluster-specific marker genes. For two given clusters, DEGs were identified by the find.markers function with the following parameters: thresh.use = 1, test.use = “roc”. For a certain gene, the roc test generated a value ranging from 0 (for ‘random’) to 1 (for ‘perfect’), representing the ‘classification power’. Genes with a fold change ≥ 2 or ≤ 0.5 and a power ≥ 0.4 were selected. The pheatmap package in R was used to plot heatmaps. Violin plots were generated using Seurat. Network enrichment analysis was performed using Metascape [24] (http://metascape.org/). The identified TFs and DEGs are listed in Additional file 4.

For Fig. 7, we selected all the TFs that regulated at least one of *Epcam*, *Vim*, *Cdh1*, *Cdh2*, and *Fn1*, as inferred from the SCENIC analysis. Among these TFs, *Grhl2*, *Hnf1b*, and *Hnf4a* tended to play important roles in regulating the epithelial cells. We then extracted the gene list that was regulated by these TFs to perform network enrichment analysis using Metascape; the top 10 enriched terms are displayed in Fig. 7c.

### Developmental pseudotime analysis

We used the Monocle2 package [43, 44] in R to determine the developmental pseudotimes of organs. Following the Monocle vignette, we used UMI count data as input and selected genes with high dispersion (more than twice the fitted dispersion) for unsupervised ordering of the cells. The default settings were used for all other parameters.

### Cell cycle analysis and identification of surface markers and transcription factors

To perform cell cycle analysis, we used a previously defined core gene set, including 43 G1/S and 54 G2/M genes [45, 46]. The average expression of each gene set was calculated as the corresponding score. Cells with a G1/S score < 2 and a G2/M score < 2 were determined to be quiescent; otherwise, they were deemed proliferative. Additionally, proliferative cells with a G2/M score > G1/S score were designated G2/M, whereas cells with a G1/S score > G2/M score were designated G1/S. TFs were selected from the 1485 TFs included in AnimalTFDB 2.0 [47], and surface markers were selected from the 261 cell surface markers collected in a previous report [48].

### Single-cell qRT-PCR

Additional single cells were collected from E11.5 liver and E10.5 brain tissues. The single-cell reverse transcription and cDNA amplification were carried out following Smart-seq2 protocol. After the first round of purification with 0.8× Ampure XP beads, the cDNA of each single cell was quantified with qPCR of the housekeeping gene (*Gapdh*) and selected genes (*Alas2*, *Slc4a1*, *Bcl11a1*, *Cd47*, and *Cd24a*).

### Immunofluorescence

The organs or embryos were fixed with 4% paraformaldehyde overnight at 4 °C and then dehydrated with 20% sucrose solution overnight at 4 °C. After that, they were embedded with Tissue-Tek® O.C.T. Compound (Sakura #4583). Sections 6 μm thick were permeabilized in Triton X-100 (0.5% in PBS) for 30 min at room temperature and incubated in blocking buffer (0.1% Triton X-100 and 10% donkey serum in PBS) for 90 min at room temperature. Sections were incubated with diluted primary antibodies overnight at 4 °C and then with diluted secondary antibodies (1:400) at room temperature for 2 h. Finally, sections were immersed in ProLong Gold antifade reagent with 4’,6-diamidino-2-phenylindole (DAPI, Invitrogen #1846939). Images were acquired using a confocal laser scanning microscope (Leica TCS SP8, Leica Microsystems, Wetzlar, Germany). The primary antibodies used in this study were mouse anti-E-cadherin (Abcam #ab76055, 1:50 dilution), rabbit anti-vimentin antibody (Abcam #ab92547, 1:50 dilution), and rabbit anti-fibronectin antibody (Abcam #ab23750, 1:50 dilution).

## Additional files

Additional file 1: **Figure S1.** Quality control of the dataset and the characteristics of clusters. **Figure S2.** Interaction between epithelial and mesenchymal cells sampled from intestine, liver, lung, and skin. **Figure S3.** Immunostaining of Cdh1, Vim, and Fn1 in E9.5 and adult liver. **Figure S4.** Comparison between definitive and primitive erythroid cells. **Figure S5.** Comparison between neuronal cells sampled from forebrain and hindbrain. **Figure S6.** Expression patterns of cells sampled from heart. **Figure S7.** Expression patterns of cells sampled from somites. (PDF 17147 kb)

Additional file 2: Barcode-RT primer information, cell cycle gene list, and E-, M- and S-score gene list. (XLSX 18 kb)

Additional file 3: Cell information and classification. (XLSX 270 kb)

Additional file 4: DEGs and top TFs among groups and clusters. (XLSX 635 kb)
