# Supplementary material for: Single-cell RNA-seq analysis unveils a prevalent epithelial/mesenchymal hybrid state during mouse organogenesis
Source: Genome Biol. 2018 Mar 14;19:31. doi: 10.1186/s13059-018-1416-2 (PMC5853091; doi:10.1186/s13059-018-1416-2)
Supplement: Supplementary file 1 — Figure S1. Quality control of the dataset and the characteristics of clusters. Figure S2. Interaction between epithelial and mesenchymal cells sampled from intestine, liver, lung, and skin. Figure S3. Immunostaining of Cdh1, Vim, and Fn1 in E9.5 and adult liver. Figure S4. Comparison between definitive and primitive erythroid cells. Figure S5. Comparison between neuronal cells sampled from forebrain and hindbrain. Figure S6. Expression patterns of cells sampled from heart. Figure S7. Expression patterns of cells sampled from somites. (PDF 17147 kb) [file 13059_2018_1416_MOESM1_ESM.pdf]

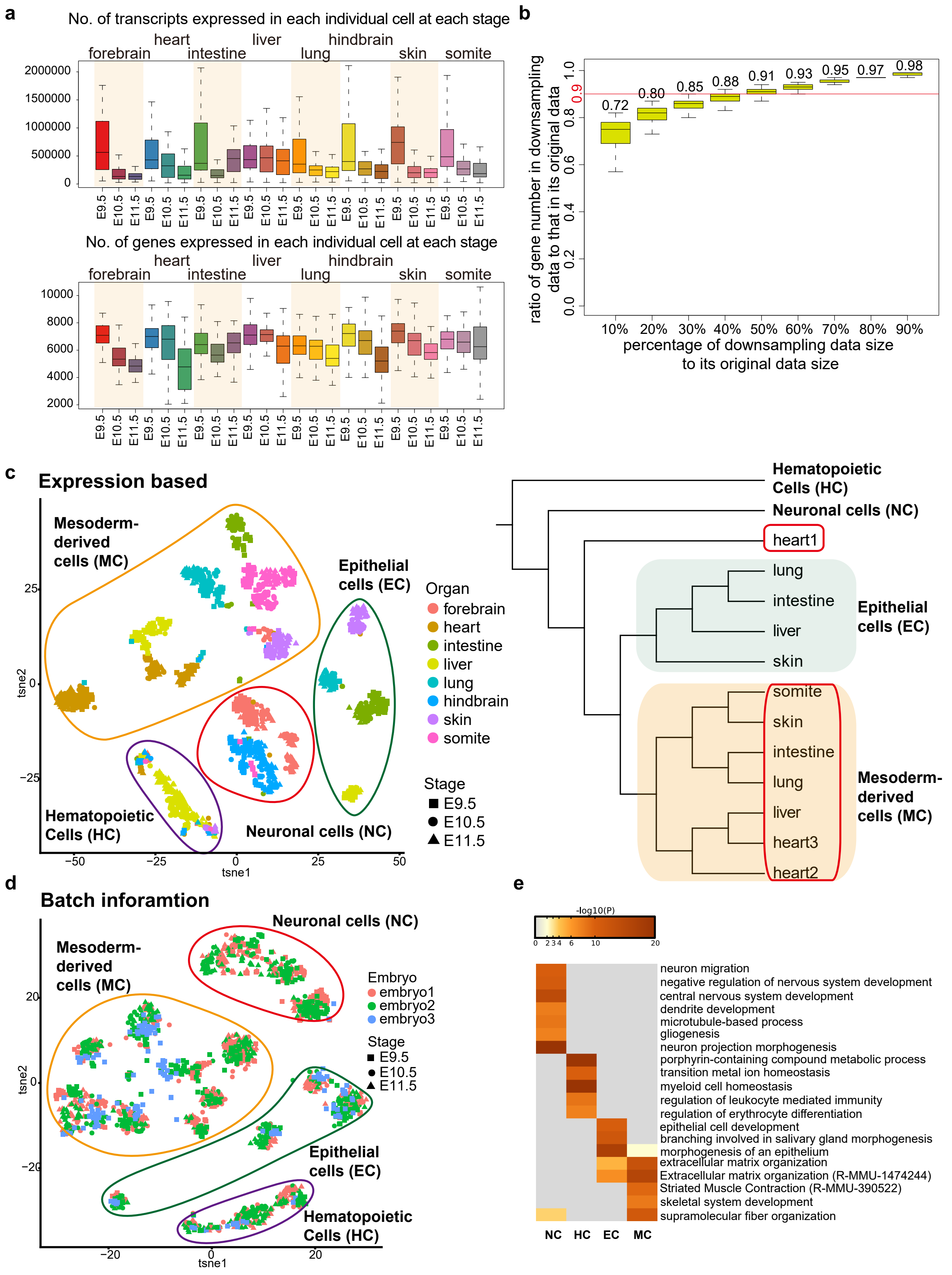

Figure S1. Quality control of the data set and the characteristics of clusters

(a) Boxplots showing the number of transcripts and the number of genes detected in the single cells from different organs. (b) Result of down-sampling analysis (see Methods section for details): the x-axis represents the percentage of the down-sampling data size to its original data size, and the y-axis represents the percentage of detected genes from the down-sampling data compared to that from the original data. The number above each box indicates the percentage of the mean gene number. (c) Expression matrix-based t-SNE plot showing the origin and embryonic stage of the cells (left). Organs are indicated by colors, and developmental stages are indicated by shapes. The major groups identified by the regulon matrix are circled and annotated. Hierarchical clustering through the expression matrix showing the relationships of cells sampled from different organs (right). Major groups identified by the regulon matrix are listed on the right. (d) Batch information mapped on the tSNE from Fig. 1b. Embryos are indicated by colors, and developmental stages are indicated by shapes. (e) Heatmaps showing enrichment of marker genes of each major group. The color key from gray to brown indicates high to low P-value, respectively.

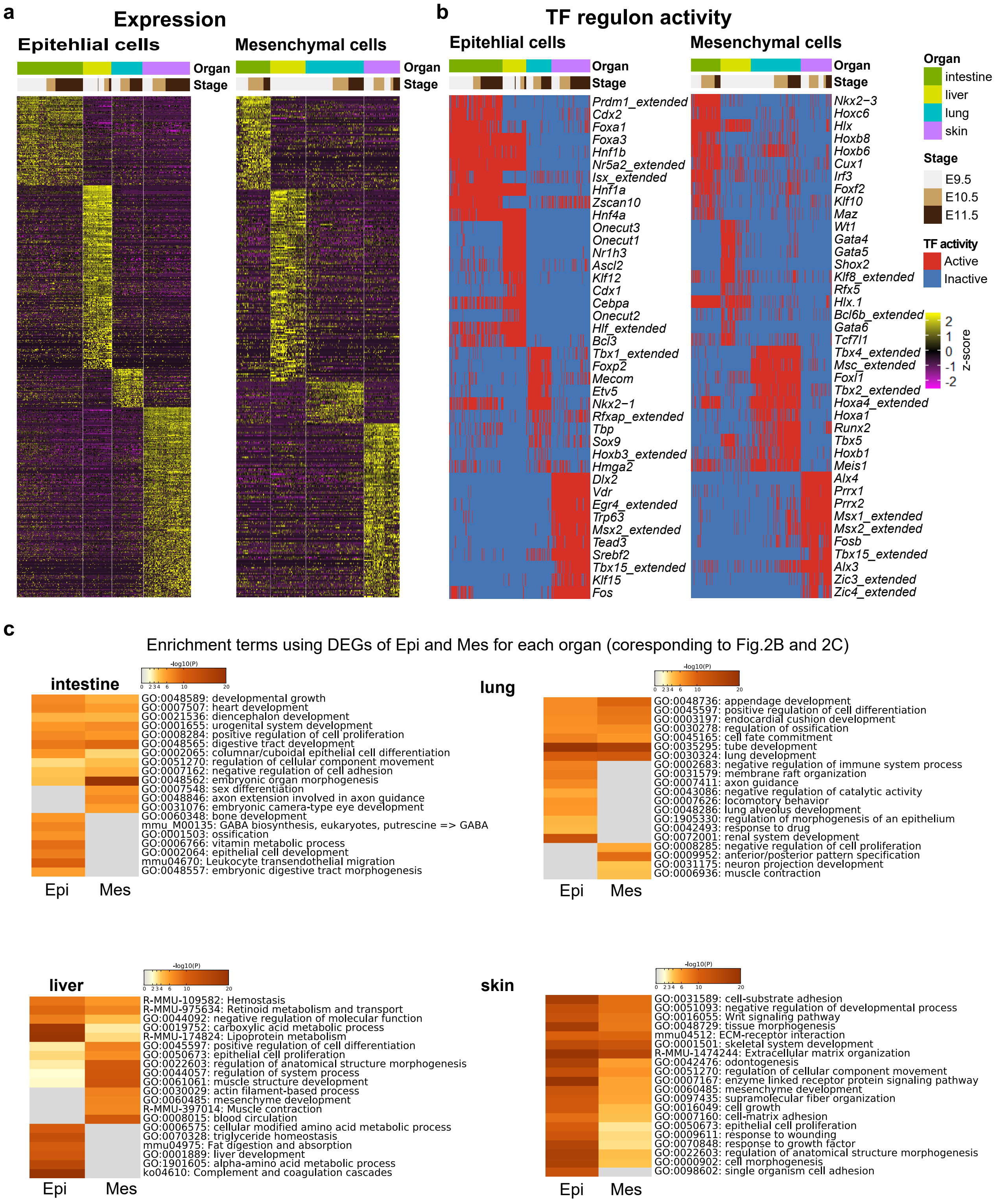

Figure S2. Interaction between epithelial and mesenchymal cells sampled from intestine, liver, lung and skin

(a) Heatmap showing all DEGs in epithelial (left) and mesenchymal (right) cells sampled from intestine, liver, lung and skin. The color key from purple to yellow indicates low to high gene expression, respectively. (b) Heatmap showing the top10 TFs in epithelial (left) and mesenchymal (right) cells sampled from intestine, liver, lung and skin. The color key from blue to red indicates low to high TF activity, respectively. (c) Heatmaps showing enrichment of epithelial and mesenchymal cells in each organ. The color key from gray to brown indicates high to low P-value, respectively.

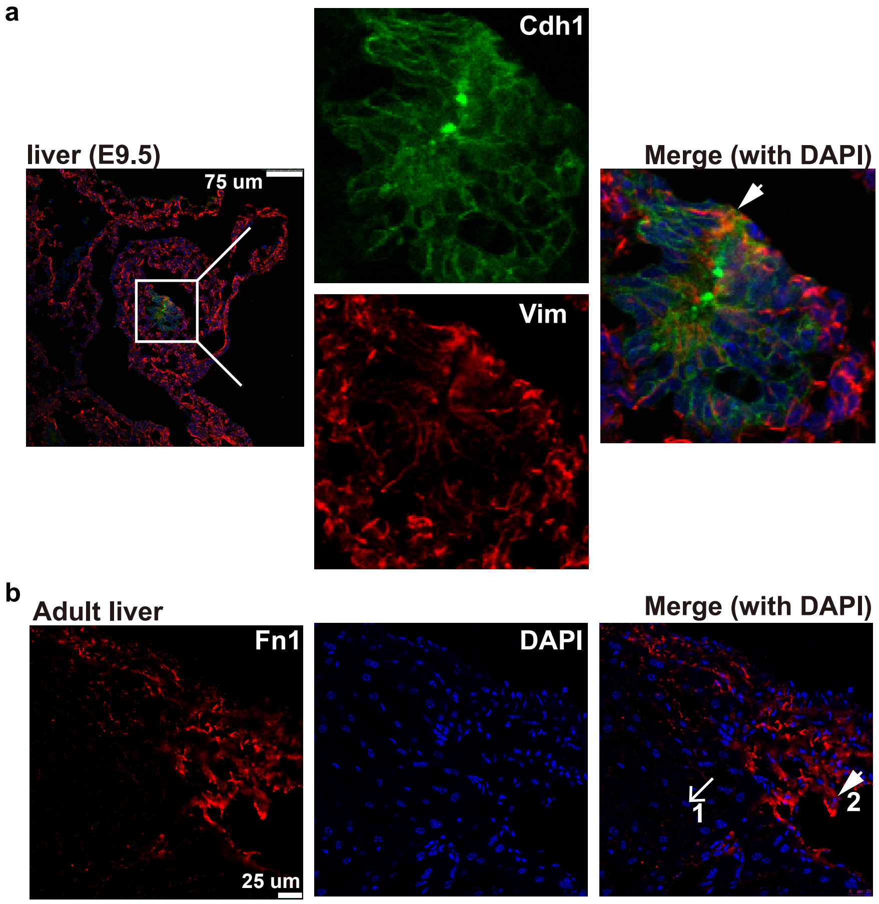

Figure S3. Immunostaining of Cdh1, Vim and Fn1 in E9.5 and adult liver
(a) Immunostaining for Cdh1 and Vim in E9.5 liver. White arrow indicates potential co-expression of Cdh1 and Vim. (b) Immunostaining for Fn1 in adult liver. Arrow 1 points to a representative cell with no staining of Fn1, while arrow 2 points to a representative cell with staining of Fn1.

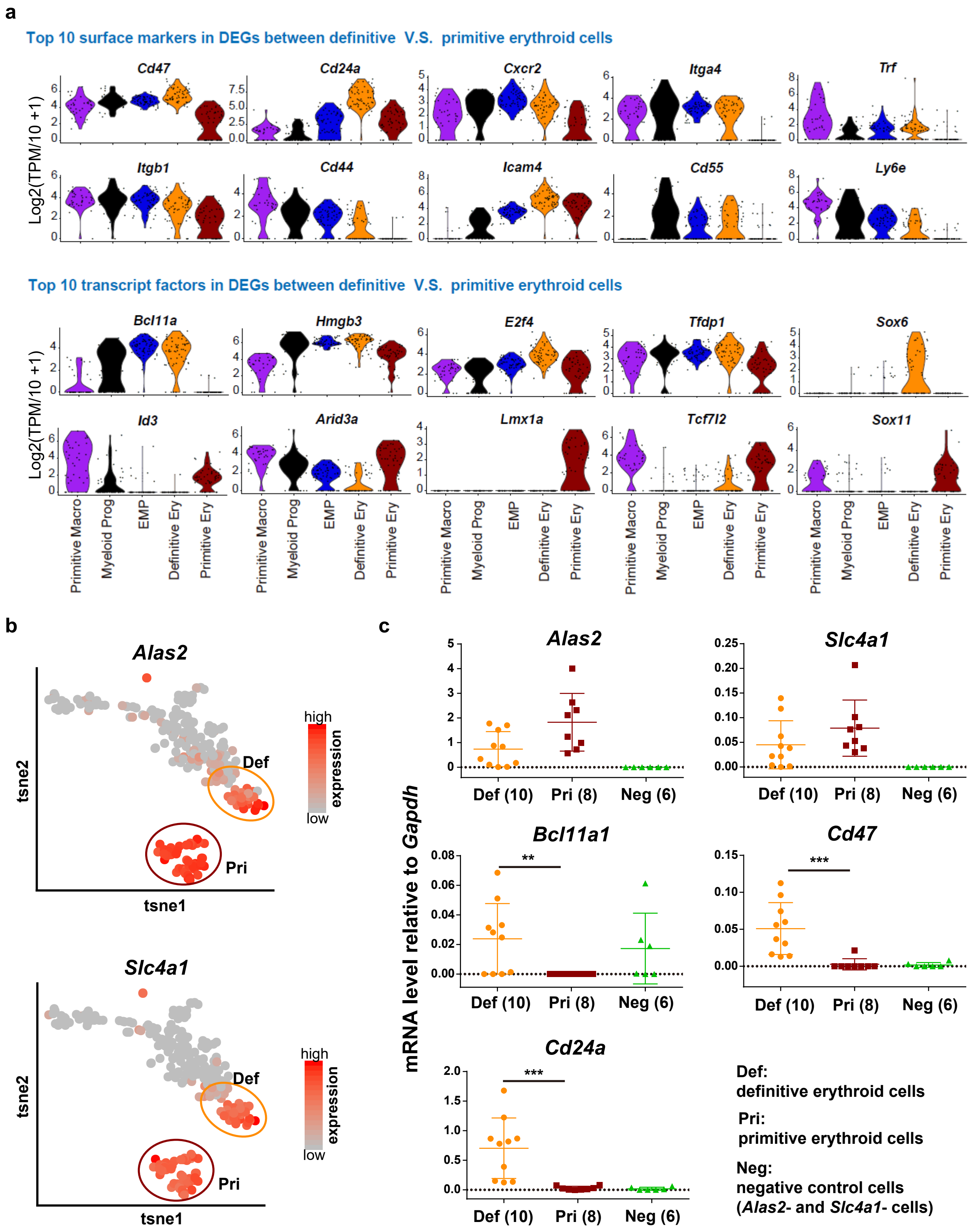

Figure S4. Comparison between definitive and primitive erythroid cells

(a) Violin plots showing the top 10 surface markers (top) and top10 TFs (bottom) among the DEGs between definitive between primitive erythroid cells. Other hematopoietic cell clusters are also displayed. (b) The relative expression of *Alas2* and *Slc4a1* mapped on the t-SNE plot from Fig. 8a. (c) Single cell qPCR validation of primitive and definitive erythroid cells. *Alas2* and *Slc4a1* were used to identify both primitive and definitive erythroid cells. And *Bcl11a1*, *Cd47*, *Cd24a* identified in Fig. S4a were used to discriminate definitive erythroid cells from primitive erythroid cells. Both *Alas2* and *Slc4a1* negative cells were regarded as negative control cells. Single cell number is displayed in the brackets. Mann-Whitney test was used to test the difference between primitive and definitive erythroid cells. ** indicates p value <0.01 and ***indicates p value < 0.001. The qPCR result showed that definitive erythroid cells were detected in E11.5 liver while primitive erythroid cells were detected in E10.5 brain tissue, which is consistent with that of single-cell RNA-seq analysis.

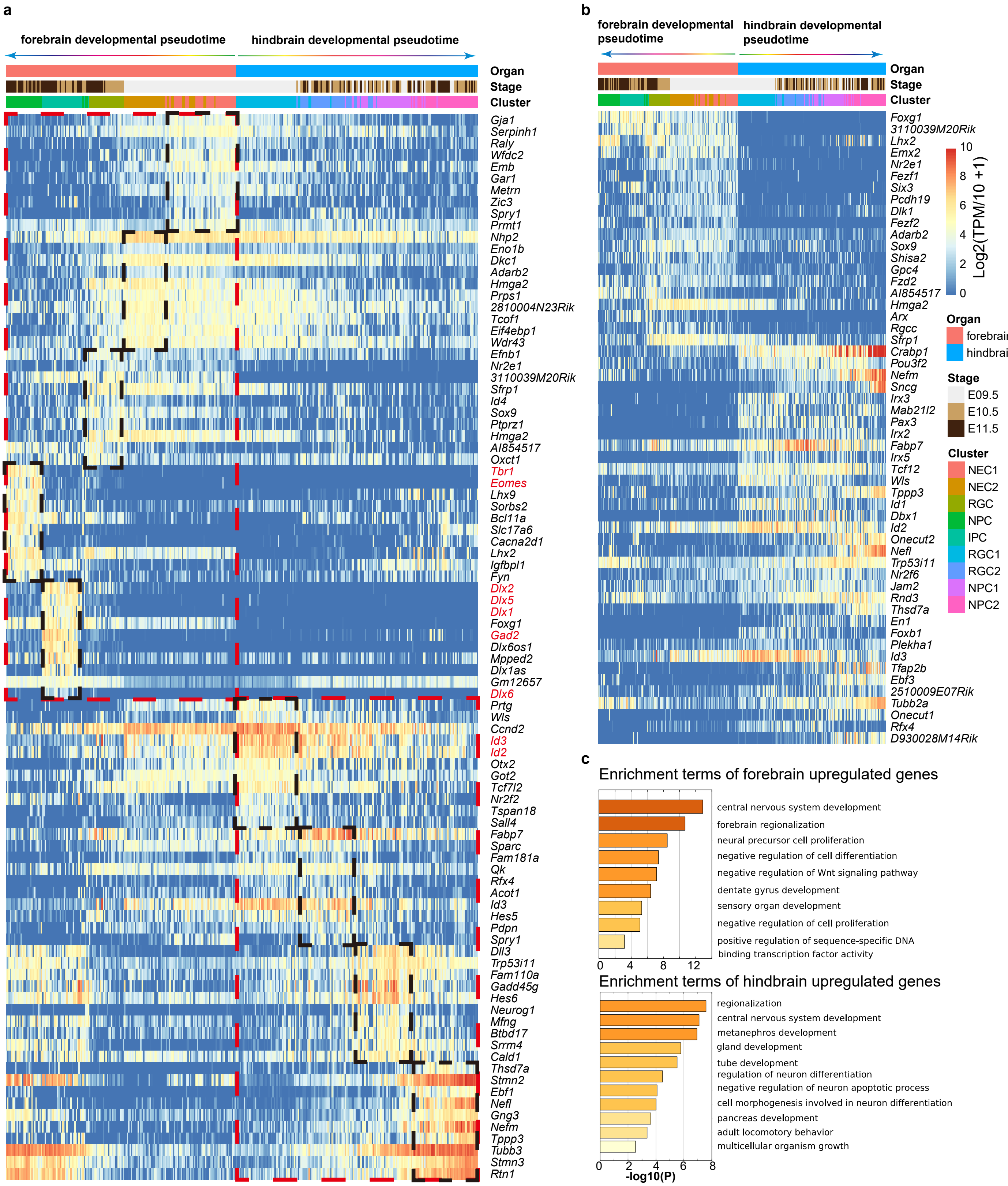

Figure S5. Comparison between neuronal cells sampled from forebrain and hindbrain

(a) Heatmap showing the top 10 DEGs in each neuronal cluster. The color key from blue to red indicates low to high gene expression, respectively. (b) Heatmap showing the top 20 DEGs between forebrain and hindbrain neuronal cells. (c) Enrichment of DEGs between forebrain and hindbrain neuronal cells.

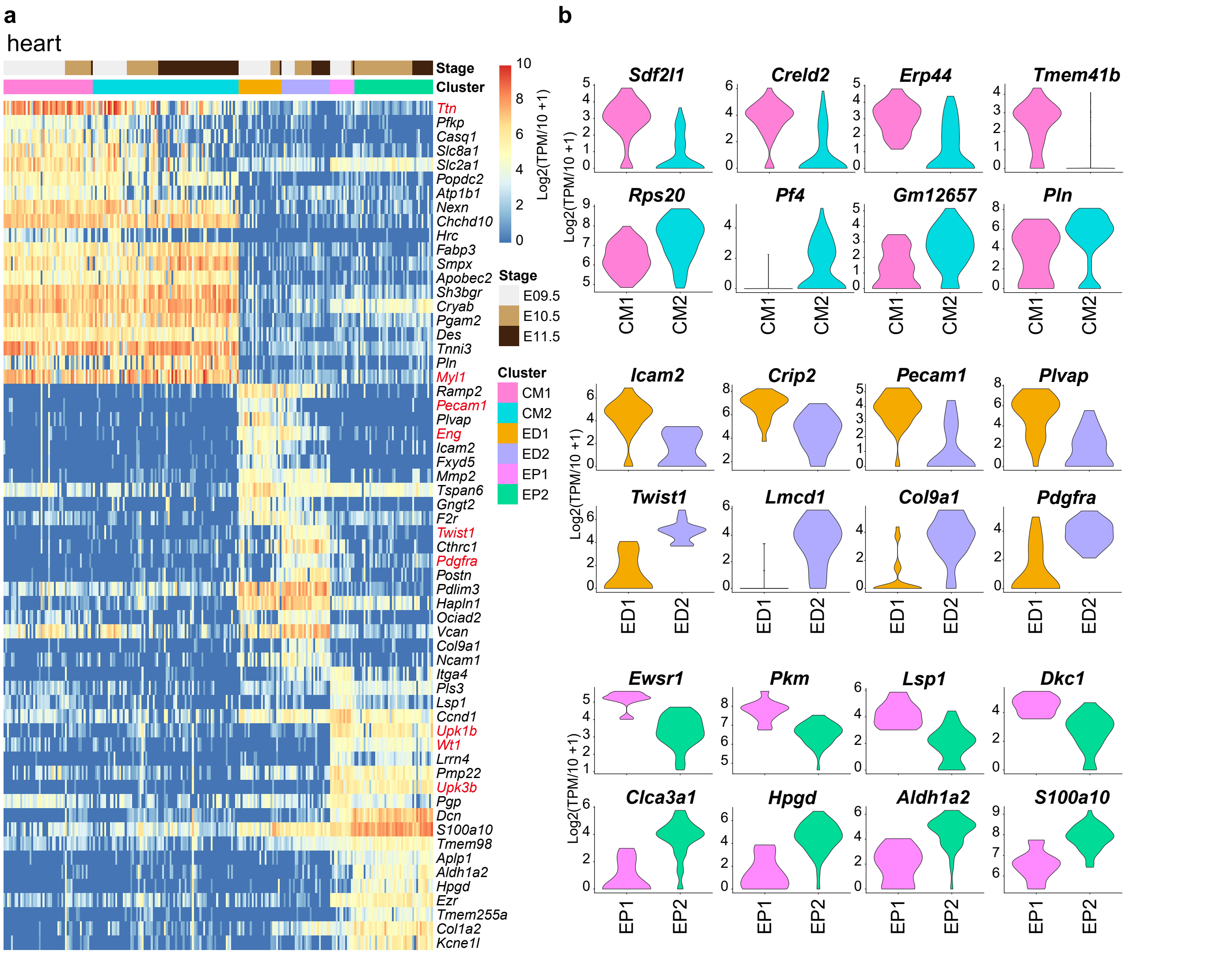

Figure S6. Expression patterns of cells sampled from heart

(a) Heatmap showing the top 10 DEGs in each heart cluster. The color key from blue to red indicates low to high gene expression, respectively.

(b) Violin plots showing the expression of selected markers between two subgroups of each cell type.

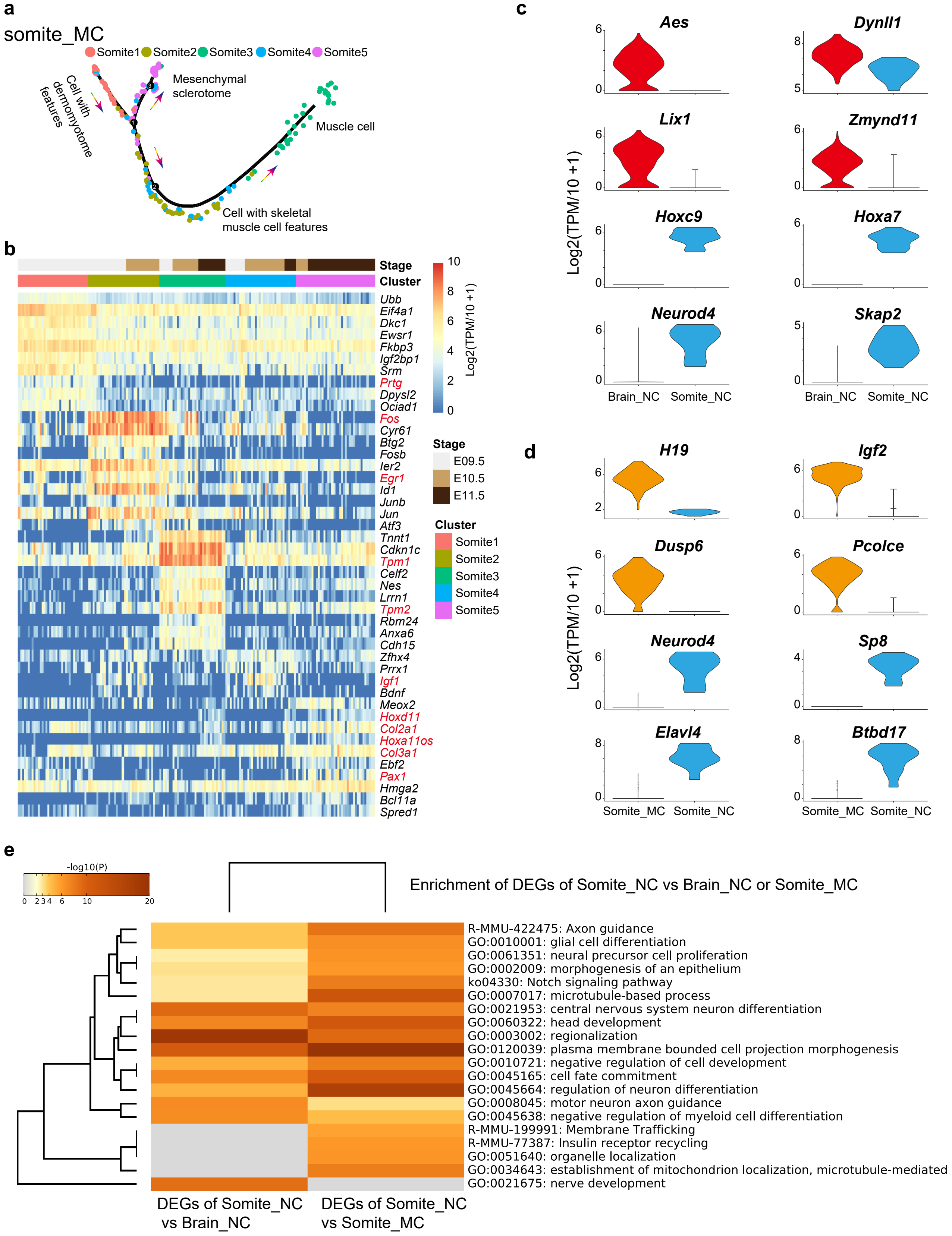

Figure S7. Expression patterns of cells sampled from somites

(a) Developmental pseudotime of somite cells inferred by Monocle2. Clusters are indicated by colors. (b) Heatmap showing the top 10 DEGs in each somite cluster. (c) Violin plots showing the expression of selected markers between somite neuronal cells and brain neuronal cells. (d) Violin plots showing the expression of selected markers between somite neuronal cells and somite mesenchymal cells. (e) Heatmap showing enrichment of DEGs of somite neuronal cells compared with brain neuronal cells.and mesenchymal cells. The color key from gray to brown indicates high to low P-value, respectively.
